# Learning interventions and training methods in health emergencies: A scoping review

**DOI:** 10.1371/journal.pone.0290208

**Published:** 2024-07-16

**Authors:** Heini Utunen, Giselle Balaciano, Elham Arabi, Anna Tokar, Aphaluck Bhatiasevi, Jane Noyes

**Affiliations:** 1 Health Emergencies Programme, Learning and Capacity Development Unit, World Health Organization, Genève, Switzerland; 2 Department of Medical and Health Sciences, Bangor University, Bangor, United Kingdom; Ladoke Akintola University of Technology Teaching Hospital: LAUTECH Teaching Hospital, NIGERIA

## Abstract

**Background:**

Keeping the health workforce and the public informed about the latest evolving health information during a health emergency is critical to preventing, detecting and responding to infectious disease outbreaks or other health emergencies. Having a well-informed, ready, willing, and skilled workforce and an informed public can help save lives, reduce diseases and suffering, and minimize socio-economic loss in affected communities and countries. Providing “just in time” support and opportunities for learning in health emergencies is much needed for capacity building. In this paper, ‘learning intervention’ refers to the provision of ad-hoc, focused, or personalized training sessions with the goal of preparing the health workers for emergencies or filling specific knowledge or skill gaps. We refer to ‘training methods’ as instructional design strategies used to teach someone the necessary knowledge and skills to perform a task.

**Methods:**

We conducted a scoping review to map and better understand what learning interventions and training methods have been used in different types of health emergencies and by whom. Studies were identified using six databases (Pubmed/Medline, Embase, Hinari, WorldCat, CABI and Web of Science) and by consulting with experts. Characteristics of studies were mapped and displayed and major topic areas were identified.

**Results:**

Of the 319 records that were included, contexts most frequently covered were COVID-19, disasters in general, Ebola and wars. Four prominent topic areas were identified: 1) Knowledge acquisition, 2) Emergency plans, 3) Impact of the learning intervention, and 4) Training methods. Much of the evidence was based on observational methods with few trials, which likely reflects the unique context of each health emergency. Evolution of methods was apparent, particularly in virtual learning. Learning during health emergencies appeared to improve knowledge, general management of the situation, quality of life of both trainers and affected population, satisfaction and clinical outcomes.

**Conclusion:**

This is the first scoping review to map the evidence, which serves as a first step in developing urgently needed global guidance to further improve the quality and reach of learning interventions and training methods in this context.

## Introduction

Learning in health emergencies can provide a foundation for building capacity for emergency preparedness and response specific to different health emergencies [1] (e.g., biological, environmental, armed conflicts, deliberate acts of terrorism, industrial accidents), especially in low- and middle-income countries where health systems need to be further strengthened [2]. A ready, willing, and able workforce is required that can be called upon in health emergencies to help save lives, reduce disease and suffering, and minimize socio-economic loss in affected communities and countries. In today’s interconnected landscape, an educated public is also needed to champion measures for a strong emergency preparedness and effective response [1]. This can be achieved by guiding, standardizing and facilitating the delivery of life-saving knowledge, first to frontline workers in health emergencies and second to the public. Learning paves the way for strengthened health literacy and understanding of health communication which, in turn, bolsters awareness and support for measures needed during health emergency events [3].

The World Health Organization (WHO) is developing guidance on *Learning in Emergencies* to provide evidence-based interventions, training methods and tools for professionals, communities and institutions to ensure quality learning provision and knowledge dissemination during health emergencies. The primary reason for developing this guidance emerged from lessons learned from the COVID-19 pandemic, which presented unique circumstances and global challenges. It became clear that ‘just in time’ learning is required to retrain and upskill large numbers of health professionals in order to launch an effective response. For example, new methods and approaches for testing and surveillance of COVID-19 at scale were proven essential to operate at the national, regional, and local level, which involved the need for rapid dissemination and uptake of new learning so that the entire health, social care and education workforce were equipped with relevant knowledge and skills to respond effectively, especially in low- and middle-income countries [4, 5].

The COVID-19 pandemic also prompted the need to integrate effective risk communication strategies to tackle the evolving information needs and misinformation or infodemic (i.e., short for information epidemic) [6] and potential spread of misinformation that could negatively impact on efforts to develop and deliver learning interventions to professionals, communities and institutions [7]. From the onset of the COVID-19 pandemic, the World Health Organization (WHO) committed to knowledge dissemination to support frontline health workers, governmental and non-governmental actors, policy makers, capacity builders and trainers as well as the public via its low-bandwidth adjusted online platform. In addition to regular updates as more research emerged, WHO intended to regularly assess the effectiveness of this initiative—that is knowledge dissemination through its online platform (OpenWHO) on all the aspects of tackling the pandemic and then including other health emergencies. Key findings indicated that employing strategies to make learning more equitable and accessible can yield better results in terms of outreach, and that learning production must be targeted for real-time events in languages spoken in outbreak impacted areas [8]. Nonetheless, learning design based on inclusive pedagogy and learning sciences, while being cognizant of barriers to accessing learning (such as poor internet connection or limited digital literacy) can optimize learning experience [9].

The second rationale for developing the Learning in Emergencies guidance is that various current guidelines tackled adult learning partially or in one dimension. WHO itself has published several frameworks and recommendations. However, none of these related guidelines covers Adult Learning in Health Emergencies. The Learning in Emergencies guidance will address a priority issue cited during the World Health Assembly (WHA), to help WHO support governments in their health-related capacity building and to reach their health learning goals. The *Learning in Emergencies* guidance will encompass the full scope of learning as preparedness, readiness, response and resilience actions in capacitation for public health emergencies.

This scoping review of literature was undertaken to inform further methodological choices and the commissioning of subsequent systematic reviews to feed into the Guidance development process.

## Objectives

The objective of this scoping review was to map the existing evidence (including qualitative, quantitative and mixed-methods peer-reviewed publications, systematic and other types of literature reviews and qualitative evidence synthesis) that have been published on the topic.

## Target population

The target population included experts and individuals in need of health information, such as the health workforce, experts and volunteers, national institutions and ministries (governmental and non-governmental actors), policymakers, academia, capacity builders and trainers, citizens and affected populations.

## Phenomenon of interest

The phenomena of interest were classified as learning interventions as specified below and health emergencies including the follow types: Biological, Environmental, Armed conflicts, Deliberate acts of terrorism and Industrial accidents.

## Intervention/Exposure

Learning interventions included:

- Continuous learning for professionals preparing for or acting in health emergencies
- Adult learning interventions and methods in emergency situations
- Professional education, training and learning
- Real time, just in time learning
- Knowledge and learning transfer from an expert organization
- Learning readiness in anticipation of and preparedness for any health hazard

Exclusion criteria:

- Professional learning
- University degrees
- Postgraduate studies

## Methods

The methodology was guided by Arksey and O’Malley’s [8] five-stage framework for scoping reviews: identifying the research question(s), identifying relevant studies, charting the data, collating, summarizing, and reporting the results. In the latter case this means reporting the results of the searches and mapping the studies and not providing any detailed analysis of included study results. In addition, principles of mixed-methods framework synthesis were used to manage diverse study designs and help extract, map, chart, categorize and summarize studies under four prominent topic areas [10]. Like McGill and colleagues in their recent scoping review on knowledge exchange in crisis settings [11], we did not go beyond Arksey and O’Malley’s scoping review methodology to undertake a synthesis of findings. This aligned with the purpose of this scoping review to identify published peer reviewed studies from which subsequent review questions could be formulated and systematic reviews commissioned. The review was reported using the relevant domains of the Preferred Reporting items for Systematic Review and Meta-Analysis for scoping reviews (PRISMA-ScR) [12].

A priori protocol was developed and published on the Open Science Framework: https://doi.org/10.17605/OSF.IO/5BK9R.

### Selecting relevant studies

Searches were carried out between 2 February and 28 February 2023, and covered sources from 2003 to the present. Key search terms were identified within three PICO (Problem/Population, Intervention, Comparison, Outcome) question components (S1 Table in S1 File). These terms were also used to identify relevant documents from which Medical Subject Heading (MeSH) or other database-specific terms and keywords could be extracted.

Key terms were searched using a free text strategy in the titles and abstracts. This allowed having a broader, more sensitive approach and eliminated the possibility of relevant items being missed. MeSH was applied to give more specific results.

The following databases were searched: Pubmed/Medline, Embase, Hinari, WorldCat, CABI and Web of Science, using predefined combinations of key search terms. To prioritize LMICs (low- and middle-income countries), filters of the selected databases were applied. The search strategy and search words are annexed (Full strategy in supplementary material). A systematic grey literature search was performed and, due to the nature and objective of the information retrieved, will be published in a separate article.

Search results were scanned for relevance and those meriting further examination were imported into Rayyan for further consideration. The search and initial screening were undertaken by GB and studies checked by other authors were included. Citations were excluded if they focused exclusively on academic settings (early education through medical school), did not have a learning intervention (for example studies addressing knowledge in unprepared professionals) or were not contextualized during or for a health emergency. News (or announcements) as well as protocols of studies/reviews that had not been completed, and other irrelevant document types were excluded, as well as non-peer reviewed articles.

Titles and abstracts were screened against the following inclusion criteria: (1) published between 2003 and 2023; (2) abstract and title written in English; (3) presenting data on the research question (date, methodology, focus on geographical low-, middle- and high-income context, type of learning/intervention and for whom, type of emergency); (4) peer-reviewed sources only. Then screening of full texts of pre-selected citations against above-mentioned criteria was performed. At this stage full texts written in other United Nations languages and Portuguese language were included as well. Processes were undertaken by GB and crossed checked by co-authors. We did not undertake double-blind processing.

### Charting the data

All the included evidence was extracted into an Excel spreadsheet and included citations were exported into Endnote. Data were extracted systematically using a standardized form that included information on the period of study, location, study population, design, research questions, key findings, and conclusions.

### Collating, summarizing and reporting results

The charted data (in the form of an evidence map) were then further analyzed, grouped and sorted, guided by the review aim and objectives. We specifically focused on developing an understanding of the available literature on the phenomena of interest and created visual displays and tables. We also identified and described four major topic areas.

## Results

**Fig 1:**
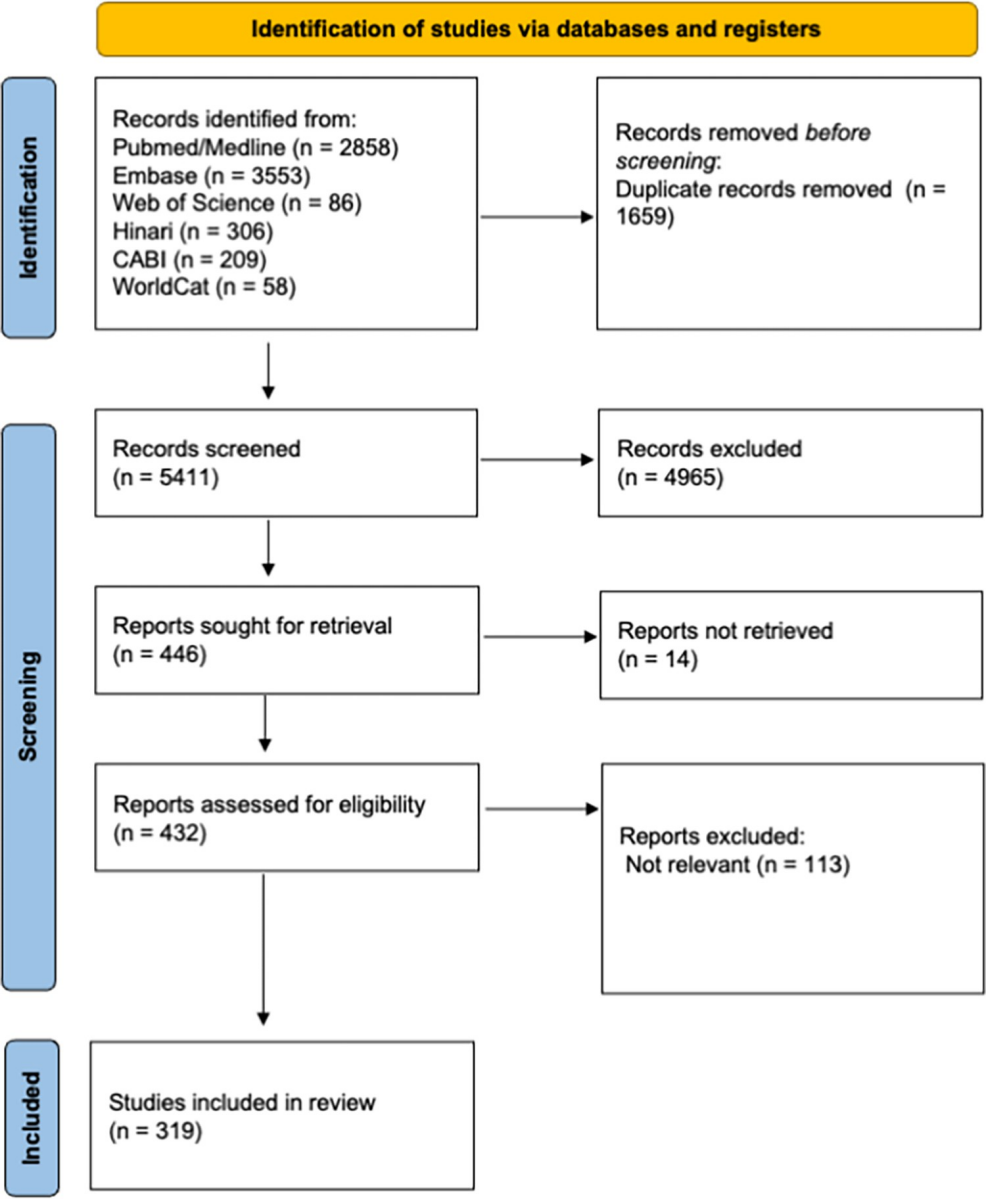
PRISMA flowchart.

A total of 6411 articles were imported into Rayyan, of which 1656 were duplicates and 4317 were excluded based on a brief scan of titles and abstracts. The remaining 446 documents were sorted by full-text access. One hundred and thirteen articles were excluded for not being relevant and fourteen did not have full text access. As shown in Fig 1, 319 articles met the inclusion criteria and were included. Full description of included studies can be found in Table 1.

**Table 1:** Description of included studies.

Author	Year	Title	Country	Disaster/Emergency	Population	Learning Method	Major topic	Study design
Panda R	2022	Evaluation of COVID-19 ECHO training program for healthcare workers in India—A Mixed-Method Study.	India	COVID-19	Health workforce	Virtual training	Knowledge evaluation	Cross-sectional
Camputaro LA	2021	Intensive competency-based training strategy in a National Hospital in times of Pandemic.	El Salvador	COVID-19	Health workforce	In-person training	Knowledge evaluation	Qualitative
Siddiqui SS;	2023	The impact of a "short-term" basic intensive care training program on the knowledge of nonintensivist doctors during the COVID-19 pandemic: An experience from a population-dense low- and middle-income country.	India	COVID-19	Health workforce	In-person training	Knowledge evaluation	Observational
Jordan P	2023	Development of a training programme for professional nurses in South Africa—An educational response to the COVID-19 pandemic.	South Africa	COVID-19	Health workforce	In-person training	Learning technique	Report
Kharel R	2022	Training program for female community volunteers to combat COVID 19 in rural Nepal.	Nepal	COVID-19	Experts and volunteers	Virtual training	Learning technique	Report
Caviglia M	2022	Response to Mass-Casualty Incidents and Outbreaks: A Prehospital Disaster Training Package Developed for the National Emergency Medical Service in Sierra Leone.	Sierra Leone	Disasters	Health workforce	In-person training	Learning technique	Report
Singh SS	2022	Training community health workers for the COVID-19 response, India.	India	COVID-19	Health workforce	In-person training	Impact	Cross-sectional
Guragai M	2020	Medical Students’ Response to the COVID-19 Pandemic: Experience and Recommendations from Five Countries.	Brazil, Nepal, the Philippines, Rwanda, and the United States.	COVID-19	Health workforce and community	Blended	Learning technique	Report
Marsh RH;	2021	Facing COVID-19 in Liberia: Adaptations of the Resilient and Responsive Health Systems Initiative.	Liberia	COVID-19	Health workforce	Virtual training	Learning technique	Report
Uttekar S	2023	Empowering Health Workers to Build Public Trust in Vaccination: Experience from the International Pediatric Association’s Online Vaccine Trust Course, 2020–2021.	International	COVID-19	Health workforce	Virtual training	Knowledge evaluation	Report
Zerdo Z;	2022	Implementation of a malaria prevention education intervention in Southern Ethiopia: a qualitative evaluation.	Etihopia	others	Health workforce	In-person training	Impact	Randomized controlled trial (RCT)
Perera N	2022	Implementation of a coronavirus disease 2019 infection prevention and control training program in a low-middle income country.	Syria	COVID-19	Health workforce	In-person training	Knowledge evaluation	Report
Cuen AJ	2022	Fighting COVID-19 and HIV through community mobilisation: lessons from an integrated approach to the Africa CDC Partnership to Accelerate COVID-19 Testing (PACT) initiative in seven countries.	Africa	COVID-19	Health workforce and community	Blended	Learning technique	Review
Malik JA;	2022	Myths and misconception of COVID-19 among hospital sanitary workers in Pakistan: Efficacy of a training program intervention.	Pakistan	COVID-19	Health workforce	Virtual training	Knowledge evaluation	Before-after
Osula VO	2022	COVID-19 advanced respiratory care educational training program for healthcare workers in Lesotho: an observational study.	Lesotho	COVID-19	Health workforce	In-person training	Knowledge evaluation	Before-after
Cianelli R	2013	Mental health training experiences among Haitian healthcare workers post-earthquake 2010.	Haiti	Earthquake	Health workforce	In-person training	Impact	Observational
Lu Y	2016	Chinese military medical teams in the Ebola outbreak of Sierra Leone.	Sierra Leone	Ebola	Military	In-person training	Learning technique	Report
Yilmaz	2021	Real time COVID-19 online learning for medical students: a massive open online course evaluation.	Turkey	COVID-19	Health workforce	Virtual training	Impact	Cross-sectional
Pek JH	2020	Teaching Disaster Site Medical Support in Indonesia.	Indonesia	Disasters	Health workforce	In-person training	Learning technique	Report
He LX	2022	Perspectives of nursing directors on emergency nurse deployment during the pandemic of COVID-19: A nationwide cross-sectional survey in mainland China	China	COVID-19	Health workforce	N/A	Emergency plans	Cross-sectional
Ray S	2021	Innovation in primary health care responses to COVID-19 in Sub-Saharan Africa.	Africa	COVID-19	Health workforce	N/A	Emergency plans	Review
Kochis M;	2021	Learning During and From a Crisis: The Student-Led Development of a COVID-19 Curriculum	International	COVID-19	Health workforce	In-person training	Learning technique	Report
Shahrin L	2022	In-person training on COVID-19 case management and infection prevention and control: Evaluation of healthcare professionals in Bangladesh.	Bangladesh	COVID-19	Health workforce	In-person training	Knowledge evaluation	Cross-sectional
Müller SA	2020	Implementation of the WHO hand hygiene strategy in Faranah regional hospital, Guinea.	Guinea	Ebola	Health workforce	In-person training	Knowledge evaluation	Before-after
Fredricks K;	2017	Community Health Workers and Disasters: Lessons Learned from the 2015 Earthquake in Nepal.	Nepal	Earthquake	Health workforce	N/A	Emergency plans	Qualitative
Eardley W	2016	Education and Ebola: initiating the cascade of emergency healthcare training.	Africa	Ebola	Health workforce	In-person training	Knowledge evaluation	Before-after
Chemali Z;	2017	Humanitarian space and well-being: effectiveness of training on a psychosocial intervention for host community-refugee interaction.	Lebanon	War	Experts and volunteers	In-person training	Impact	Before-after
Jaguga F	2020	Mental health response to the COVID-19 pandemic in Kenya: a review.	Kenya	COVID-19	Health workforce and community	Virtual training	Emergency plans	Review
Ntahobakurira I;	2011	The Rwanda Field Epidemiology and Laboratory Training	Rwanda	others	Health workforce	In-person training	Learning technique	Report
Soeters HM	2018	Infection prevention and control training and capacity building during the Ebola epidemic in Guinea.	Guinea	Ebola	Health workforce	In-person training	Knowledge evaluation	Before-after
Stander M;	2011	Hospital disaster planning in the Western cape, South Africa.	South Africa	Disasters	Health workforce	N/A	Emergency plans	Cross-sectional
Naghavi Alhosseini	2018	Earthquake in the city: using real life gamification model for teaching professional commitment in high school students.	Iran	Earthquake	Citizens and affected population	Virtual training	Emergency plans	Before-after
Setiawan E	2021	Evaluating knowledge and skill in surgery clerkship during covid 19 pandemics: A single-center experience in Indonesia.	Indonesia	COVID-19	Health workforce	In-person training	Knowledge evaluation	Cross-sectional
Dababnah S	2019	Feasibility of a trauma-informed parent-teacher cooperative training program for Syrian refugee children with autism.	Turkey	War	Citizens and affected population	In-person training	Impact	Before-after
Hawkes M;	2009	Use and limitations of malaria rapid diagnostic testing by community health workers in war-torn Democratic Republic of Congo.	Congo	others	Health workforce	In-person training	Knowledge evaluation	Before-after
Bustamante ND	2020	The Haiti Humanitarian Response Course: A Novel Approach to Local Responder Training in International Humanitarian Response.	Haiti	Disasters	Health workforce	Virtual training	Learning technique	Report
Wuthisuthimethawee P	2022	How the ARCH Project Could Contribute to Strengthening ASEAN Regional Capacities on Disaster Health Management (DHM).	ASEAN Member States	Disasters	Health workforce	Simulation	Learning technique	Report
Burlew R	2014	Assessing the relevance, efficiency, and sustainability of HIV/AIDS in-service training in Nigeria.	Nigeria	others	Health workforce	Virtual training	Emergency plans	Report
Thomas JJ;	2022	Participatory Workshop-Based Intervention for Better Preparedness and Awareness About Disaster Management Among Accredited Social Health Activists in India: A Brief Report.	India	Disasters	Health workforce	Simulation	Knowledge evaluation	Before-after
Joseph JK	2012	Lay health workers and HIV care in rural Lesotho: a report from the field.	Lesotho	others	Health workforce	In-person training	Knowledge evaluation	Cross-sectional
Patel U;	2015	Ebola Outbreak in Nigeria: Increasing Ebola Knowledge of Volunteer Health Advisors.	Nigeria	Ebola	Experts and volunteers	In-person training	Knowledge evaluation	Before-after
Çiçek A	2020	Combat medic course: evaluation of trainees’ perception of learning and academic-self perception.	Turkey	War	Health workforce	In-person training	Impact	Before-after
Sonenthal PD	2022	Applying the WHO course to train emergency and inpatient healthcare workers in Sierra Leone early in the COVID-19 outbreak.	Sierra Leone	COVID-19	Health workforce	In-person training	Knowledge evaluation	Before-after
Macfarlane C;	2006	Training of disaster managers at a masters degree level: from emergency care to managerial control.	South Africa	Disasters	Health workforce	Academic training	Learning technique	Report
Kang HM	2021	Development of a Medical Support Training Program for Disaster Management in Indonesia: A Hospital Disaster Medical Support Program for Indonesia.	Indonesia	Disasters	Health workforce	Simulation	Learning technique	Report
Magaña-Valladares L	2018	A MOOC (massive online open course) as an immediate strategy to train health personnel in the cholera outbreak in Mexico.	Mexico	Cholera	Health workforce	Virtual training	Learning technique	Report
de Morais Pinto R	2021	Analyzing the reach of public health campaigns based on multidimensional aspects: the case of the syphilis epidemic in Brazil.	Brazil	others	Citizens and affected population	N/A	Emergency plans	Before-after
Engelbrecht B	2021	Prioritizing people and rapid learning in times of crisis: A virtual learning initiative to support health workers during the COVID-19 pandemic.	South Africa	COVID-19	Health workforce	Virtual training	Learning technique	Report
Zhou M	2020	Research on the individualized short-term training model of nurses in emergency isolation wards during the outbreak of COVID-19.	China	COVID-19	Health workforce	Blended	Impact	Before-after
Leitch L	2009	A case for using biologically-based mental health intervention in post-earthquake china: evaluation of training in the trauma resiliency model.	China	Earthquake	Health workforce	In-person training	Impact	Report
Ma D	2021	Does theme game-based teaching promote better learning about disaster nursing than scenario simulation: A randomized controlled trial.	China	Disasters	Health workforce	Virtual training	Knowledge evaluation	RCT
Lee PH	2018	The effectiveness of an on-line training program for improving knowledge of fire prevention and evacuation of healthcare workers: A randomized controlled trial.	China	Disasters	Health workforce	Virtual training	Emergency plans	RCT
Davidson PM	2021	Global digital social learning as a strategy to promote engagement in the era of COVID-19.	International	COVID-19	Health workforce	Virtual training	Learning technique	Observational
Gul S	2008	Multitasking a telemedicine training unit in earthquake disaster response: paraplegic rehabilitation assessment.	Pakistan	Earthquake	Citizens and affected population	Virtual training	Learning technique	Before-after
Ng YM	2020	Coronavirus disease (COVID-19) prevention: Virtual classroom education for hand hygiene.	Hong Kong	COVID-19	Health workforce	Virtual training	Learning technique	Report
Patel	2022	Simulation-based ventilatory training for the caregivers at primary and rural health care workers in Central India for dealing with COVID-19 pandemic: recommendations.	India	COVID-19	Health workforce	Simulation	Learning technique	Report
Mutabaruka E	2011	The West Africa Field Epidemiology and Laboratory Training Program, a strategy to improve disease surveillance and epidemic control in West Africa.	Africa	others	Health workforce	In-person training	Learning technique	Opinion
Sommerland N	2020	Reducing HIV- and TB-Stigma among healthcare co-workers in South Africa: Results of a cluster randomised trial.	South Africa	others	Health workforce	In-person training	Knowledge evaluation	RCT
Furkan Dağcioğlu B	2020	Social adaptation status of Syrian refugee physicians living in Turkey.	Syria	War	Health workforce	N/A	Impact	Cross-sectional
Kuhls DA	2017	Basic Disaster Life Support (BDLS) Training Improves First Responder Confidence to Face Mass-Casualty Incidents in Thailand.	Thailand	Disasters	Health workforce	In-person training	Impact	Before-after
Feldman M	2021	Community health worker knowledge, attitudes and practices towards COVID-19: Learnings from an online cross-sectional survey using a digital health platform, UpSCALE, in Mozambique.	Mozambique	COVID-19	Health workforce	Virtual training	Knowledge evaluation	Cross-sectional
Dunin-Bell O	2018	What do They Know? Guidelines and Knowledge Translation for Foreign Health Sector Workers Following Natural Disasters.	International	Disasters	Health workforce	N/A	Emergency plans	Review
Gunnlaugsson G	2019	Tiny Iceland’ preparing for Ebola in a globalized world.	Iceland	Ebola	Health workforce	In-person training	Knowledge evaluation	Qualitative
El-Khani A	2021	Enhancing Teaching Recovery Techniques (TRT) with Parenting Skills: RCT of TRT + Parenting with Trauma-Affected Syrian Refugees in Lebanon Utilising Remote Training with Implications for Insecure Contexts and COVID-19.	Syria	War	Citizens and affected population	Virtual training	Impact	RCT
Liu L	2012	Zero Health Worker Infection: Experiences From the China Ebola Treatment Unit During the Ebola Epidemic in Liberia.	Liberia	Ebola	Health workforce	In-person training	Knowledge evaluation	Report
James LE	2020	Integrating mental health and disaster preparedness in intervention: a randomized controlled trial with earthquake and flood-affected communities in Haiti.	Haiti	Earthquake	Citizens and affected population	In-person training	Impact	RCT
Chua	2008	Building partnerships to address the HIV epidemic.	Singapore	others	Health workforce	In-person training	Impact	Report
Cruz-Vega	2016	Experience in training in emergencies, Division of Special Projects in Health, Instituto Mexicano del Seguro Social.	Mexico	Disasters	Health workforce and community	Blended	Emergency plans	Report
Van Heng	2008	Non-doctors as trauma surgeons? A controlled study of trauma training for non-graduate surgeons in rural Cambodia.	Cambodia	War	Experts and volunteers	In-person training	Emergency plans	Before-after
Talisuna AO	2020	The COVID-19 pandemic: broad partnerships for the rapid scale up of innovative virtual approaches for capacity building and credible information dissemination in Africa.	Africa	COVID-19	Health workforce	Virtual training	Learning technique	Report
Ren	2017	Experiences in disaster-related mental health relief work: An exploratory model for the interprofessional training of psychological relief workers.	China	Earthquake	Health workforce	In-person training	Impact	Qualitative
Najafi Ghezeljeh T;	2019	Effect of education using the virtual social network on the knowledge and attitude of emergency nurses of disaster preparedness: A quasi-experiment study.	Iran	Disasters	Health workforce	Virtual training	Knowledge evaluation	Before-after
Hou	2018	Disaster Medicine in China: Present and Future.	China	Disasters	Health workforce	In-person training	Emergency plans	Report
McQuilkin	2017	Academic Medical Support to the Ebola Virus Disease Outbreak in Liberia.	Africa	Ebola	Health workforce	In-person training	Emergency plans	Report
Hemingway-Foday JJ;	2020	Lessons Learned from Reinforcing Epidemiologic Surveillance During the 2017 Ebola Outbreak in the Likati District, Democratic Republic of the Congo.	Congo	Ebola	Health workforce	In-person training	Emergency plans	Report
Yao K	2010	Ensuring the quality of HIV rapid testing in resource-poor countries using a systematic approach to training.	Africa	others	Health workforce	In-person training	Learning technique	Report
Bodas	2022	Training Package for Emergency Medical Teams Deployed to Disaster Stricken Areas: Has ’TEAMS’ Achieved its Goals?	Italy	Disasters	Health workforce	In-person training	Knowledge evaluation	Before-after
Bemah P	2019	Strengthening healthcare workforce capacity during and post Ebola outbreaks in Liberia: an innovative and effective approach to epidemic preparedness and response.	Liberia	Ebola	Health workforce	In-person training	Knowledge evaluation	Report
Oji MO	2018	Implementing infection prevention and control capacity building strategies within the context of Ebola outbreak in a "Hard-to-Reach" area of Liberia.	Liberia	Ebola	Health workforce	In-person training	Knowledge evaluation	Report
Najarian	2004	Disaster intervention: long-term psychosocial benefits in Armenia	Armenia	Earthquake	Health workforce	In-person training	Emergency plans	Opinion
Wang	2021	The effectiveness of E-learning in continuing medical education for tuberculosis health workers: a quasi-experiment from China.	China	others	Health workforce	Virtual training	Knowledge evaluation	Before-after
Limpakarnjanarat	2007	Long-term capacity-building in public health emergency preparedness in Thailand—short report.	Thailand	Disasters	Health workforce	N/A	Emergency plans	Report
Djalali	2009	A fundamental, national, medical disaster management plan: an education-based model.	Iran	Earthquake	Health workforce	In-person training	Knowledge evaluation	Before-after
Carlos	2015	Hospital preparedness for Ebola virus disease: a training course in the Philippines.	Philippines	Ebola	Health workforce	In-person training	Knowledge evaluation	Before-after
Cherian	2004	Essential emergency surgical, procedures in resource-limited facilities: a WHO workshop in Mongolia.	China	Disasters	Health workforce	Blended	Learning technique	Report
Welton-Mitchell	2018	An integrated approach to mental health and disaster preparedness: a cluster comparison with earthquake affected communities in Nepal.	Nepal	Earthquake	Citizens and affected population	N/A	Impact	Before-after
Wang	2010	Improving emergency preparedness capability of rural public health personnel in China.	China	Disasters	Health workforce	In-person training	Knowledge evaluation	Before-after
Vijaykumar	2006	Psychosocial interventions after tsunami in Tamil Nadu, India.	India	Disasters	Experts and volunteers	N/A	Impact	Report
Koca	2020	The effect of the disaster management training program among nursing students.	Turkey	Disasters	Health workforce	In-person training	Impact	RCT
Zhang	2021	Effect of virtual reality simulation training on the response capability of public health emergency reserve nurses in China: a quasiexperimental study.	China	COVID-19	Health workforce	Virtual training	Knowledge evaluation	RCT
Rosa	2021	A Virtual Coaching Workshop for a Nurse-Led Community-Based Palliative Care Team in Liberia, West Africa, to Promote Staff Well-Being During COVID-19.	Liberia	COVID-19	Health workforce	Virtual training	Impact	Before-after
Rajasingham	2011	Cholera prevention training materials for community health workers, Haiti, 2010–2011.	Haiti	Cholera	Health workforce	In-person training	Learning technique	Report
Shin YA	2018	The Effectiveness of International Non-Governmental Organizations’ Response Operations during Public Health Emergency: Lessons Learned from the 2014 Ebola Outbreak in Sierra Leone.	Sierra Leone	Ebola	Health workforce and community	N/A	Learning technique	Report
Ripp JA	2012	The response of academic medical centers to the 2010 Haiti earthquake: the Mount Sinai School of Medicine experience.	Haiti	Earthquake	Health workforce	In-person training	Knowledge evaluation	Report
Maduka	2015	Ethical challenges of containing Ebola: the Nigerian experience.	Nigeria	Ebola	Health workforce	In-person training	Emergency plans	Report
Olu O	2018	What should the African health workforce know about disasters? Proposed competencies for strengthening public health disaster risk management education in Africa.	Africa	Disasters	Health workforce	Academic training	Emergency plans	Review
Math	2006	Tsunami: psychosocial aspects of Andaman and Nicobar islands. Assessments and intervention in the early phase.	India	Disasters	Health workforce	In-person training	Emergency plans	Report
Chamane	2022	The effect of a mobile-learning curriculum on improving compliance to quality management guidelines for HIV rapid testing services in rural primary healthcare clinics, KwaZulu-Natal, South Africa: a quasi-experimental study.	South Africa	others	Health workforce	Virtual training	Knowledge evaluation	Before-after
Bazeyo	2013	Regional approach to building operational level capacity for disaster planning: the case of the Eastern Africa region.	Africa	Disasters	Health workforce	N/A	Emergency plans	Report
Yi	2018	Developing and implementing a global emergency medicine course: Lessons learned from Rwanda.	Rwanda	Disasters	Health workforce	In-person training	Learning technique	Report
Hébert	2020	Video as a public health knowledge transfer tool in Burkina Faso: A mixed evaluation comparing three narrative genres.	Burquina Faso	others	Health workforce	Virtual training	Learning technique	Before-after
Orach	2013	Use of the Automated Disaster and Emergency Planning Tool in developing district level public health emergency operating procedures in three East African countries.	Africa	Disasters	National Institutions	Virtual training	Emergency plans	Report
Bazeyo	2015	Ebola a reality of modern Public Health; need for Surveillance, Preparedness and Response Training for Health Workers and other multidisciplinary teams: a case for Uganda.	Uganda	Ebola	Health workforce and community	Blended	Emergency plans	Report
Sharara-Chami	2020	In Situ Simulation: An Essential Tool for Safe Preparedness for the COVID-19 Pandemic	Lebanon	COVID-19	Health workforce	Simulation	Impact	Before-after
El-Bahnasawy	2014	Selected infectious disease disasters for nursing staff training at Egyptian Eastern Border	Egypt	others	Health workforce	N/A	Knowledge evaluation	Before-after
Leow	2012	Mass casualty incident training in a resource-limited environment.	Sierra Leone	Disasters	Health workforce	In-person training	Knowledge evaluation	Before-after
Olness	2005	Training of health care professionals on the special needs of children in the management of disasters: experience in Asia, Africa, and Latin America.	International	Disasters	Health workforce	In-person training	Impact	Report
Otu	2016	Using a mHealth tutorial application to change knowledge and attitude of frontline health workers to Ebola virus disease in Nigeria: a before-and-after study	Nigeria	Ebola	Health workforce	Virtual training	Knowledge evaluation	Before-after
Orach	2013	Performance of district disaster management teams after undergoing an operational level planners’ training in Uganda.	Uganda	Disasters	Health workforce	N/A	Emergency plans	Report
Pérez-Manchón	2015	[Telemedicine, a medical social network for humanitarian aid between Spain and Cameroon].	Cameroon	Disasters	Health workforce	Virtual training	Learning technique	Report
Meade	2007	A deployable telemedicine capability in support of humanitarian operations.	Africa	Disasters	Health workforce	Virtual training	Learning technique	Report
Findyartini	2021	Supporting newly graduated medical doctors in managing COVID-19: An evaluation of a Massive Open Online Course in a limited-resource setting.	Indonesia	COVID-19	Health workforce	Virtual training	Impact	Before-after
Hess	2004	Development of emergency medical services in Guatemala.	Guatemala	Disasters	Experts and volunteers	N/A	Emergency plans	Report
Yamada	2007	Interdisciplinary problem-based learning as a method to prepare Micronesia for public health emergencies.	Hawaii	Disasters	Health workforce	N/A	Emergency plans	Report
Kizakevich	2007	Virtual simulation-enhanced triage training for Iraqi medical personnel.	Iraq	Disasters	Health workforce	In-person training	Learning technique	Report
O’Reilly G	2008	In the wake of Sri Lanka’s tsunami: the health for the south capacity-building project.	Sri Lanka	Disasters	Health workforce	N/A	Emergency plans	Report
Sullivan J	2021	The Impact of Simulation-Based Education on Nurses’ Perceived Predeployment Anxiety During the COVID-19 Pandemic Within the Cultural Context of a Middle Eastern Country.	Qatar	COVID-19	Health workforce	Simulation	Impact	Before-after
Tegegne MD	2022	Use of social media for COVID-19-related information and associated factors among health professionals in Northwest Ethiopia: A cross-sectional study.	Ethiopia	COVID-19	Health workforce	Virtual training	Knowledge evaluation	Cross-sectional
Jafree	2022	WhatsApp-Delivered Intervention for Continued Learning for Nurses in Pakistan During the COVID-19 Pandemic: Results of a Randomized-Controlled Trial.	Pakistan	COVID-19	Health workforce	Virtual training	Knowledge evaluation	RCT
Leichner A;	2021	Mental health integration in primary health services after the earthquake in Nepal: a mixed-methods program evaluation.	Nepal	Earthquake	Health workforce and community	In-person training	Knowledge evaluation	Cross-sectional
Ng	2009	China-Australia training on psychosocial crisis intervention: response to the earthquake disaster in Sichuan.	China	Earthquake	Health workforce	In-person training	Impact	Before-after
AlAssaf	2022	Challenges in Pandemic Disaster Preparedness: Experience of a Saudi Academic Medical Center.	Saudi Arabia	COVID-19	Health workforce	Blended	Emergency plans	Report
Button GJ	2022	Utilizing a "Crawl, Walk, Run" Training Model to Enhance Field Sanitation Capabilities for Peacekeeping Forces: A Recommendation for the Department of Defense Global Health Engagement Enterprise.	Senegal	COVID-19	Military	Mixed	Learning technique	Report
Oliveira	2020	Personal Protective Equipment in the coronavirus pandemic: training with Rapid Cycle Deliberate Practice.	Brazil	COVID-19	Health workforce	Simulation	Knowledge evaluation	Report
Morton Hamer MJ	2019	Enhancing Global Health Security: US Africa Command’s Disaster Preparedness Program.	Africa	Disasters	Health workforce	In-person training	Emergency plans	Report
Khan JA	2020	Impact of multi-professional simulation-based training on perceptions of safety and preparedness among health workers caring for coronavirus disease 2019 patients in Pakistan.	Pakistan	COVID-19	Health workforce	Simulation	Knowledge evaluation	Before-after
Jordans MJ	2012	Evaluation of a brief training on mental health and psychosocial support in emergencies: a pre- and post-assessment in Nepal.	Nepal	disasters	Health workforce	In-person training	Knowledge evaluation	Before-after
Lubogo M	2015	Ebola virus disease outbreak; the role of field epidemiology training programme in the fight against the epidemic, Liberia, 2014.	Liberia	Ebola	Health workforce	In-person training	Learning technique	Report
Lin L;	2014	The public health system response to the 2008 Sichuan province earthquake: a literature review and interviews.	China	Earthquake	National Institutions	N/A	Emergency plans	Report
Xia	2020	Evaluating the effectiveness of a disaster preparedness nursing education program in Chengdu, China.	China	Disasters	Health workforce	In-person training	Knowledge evaluation	RCT
Bajow N;	2022	Assessment of the effectiveness of a course in major chemical incidents for front line health care providers: a pilot study from Saudi Arabia.	Saudi Arabia	others	Health workforce	Simulation	Learning technique	Before-after
Saghafinia M	2009	Effect of the rural rescue system on reducing the mortality rate of landmine victims: a prospective study in Ilam Province, Iran.	Iran	others	Health workforce	In-person training	Knowledge evaluation	Observational
He	2021	Practice in Information Technology Support for Fangcang Shelter Hospital during COVID-19 Epidemic in Wuhan, China.	China	COVID-19	Health workforce	Virtual training	Impact	Report
Kenar	2006	Medical preparedness against chemical and biological incidents for the NATO Summit in Istanbul and lessons learned.	Turkey	Disasters	Health workforce	In-person training	Emergency plans	Report
Salita C	2019	Development, implementation, and evaluation of a lay responder disaster training package among school teachers in Angeles City, Philippines: using Witte’s behavioral model.	Philippines	Disasters	Experts and volunteers	In-person training	Knowledge evaluation	Before-after
Tauxe RV;	2011	Rapid development and use of a nationwide training program for cholera management, Haiti, 2010.	Haiti	Cholera	Health workforce	In-person training	Learning technique	Before-after
Werdhani RA	2022	A COVID-19 self-isolation monitoring module for undergraduate medical students: Linking learning and service needs during the pandemic surge in Indonesia.	Indonesia	COVID-19	Health workforce	Virtual training	Impact	Report
Alshiekhly U	2015	Facebook as a learning environment for teaching medical emergencies in dental practice.	Syria	others	Health workforce	Virtual training	Impact	Cross-sectional
Cai W;	2022	Doctor of Public Health-Crisis Management and COVID-19 Prevention and Control: A Case Study in China.	China	COVID-19	Health workforce	In-person training	Emergency plans	Report
Hageman	2016	Infection Prevention and Control for Ebola in Health Care Settings—West Africa and United States.	Africa	Ebola	Health workforce	In-person training	Emergency plans	Report
Pang	2009	Pilot training program for developing disaster nursing competencies among undergraduate students in China.	China	Disasters	Health workforce	In-person training	Knowledge evaluation	Before-after
Brisebois	2011	The Role 3 Multinational Medical Unit at Kandahar Airfield 2005–2010.	Afghanistan	War	Health workforce	Simulation	Emergency plans	Report
Gertler M	2018	West Africa Ebola outbreak—immediate and hands-on formation: the pre-deployment training program for frontline aid workers of the German Red Cross, other aid organizations, and the German Armed Forces, Wuerzburg, Germany 2014/15	Africa	Ebola	Health workforce	In-person training	Impact	Report
Alim S	2015	Evaluation of disaster preparedness training and disaster drill for nursing students.	Indonesia	Disasters	Health workforce	In-person training	Knowledge evaluation	Before-after
Boon	2009	The impact of a community-based pilot health education intervention for older people as caregivers of orphaned and sick children as a result of HIV and AIDS in South Africa.	South Africa	others	Citizens and affected population	In-person training	Impact	Report
Subedi	2018	The Health Sector Response to the 2015 Earthquake in Nepal.	Nepal	Earthquake	Health workforce	In-person training	Emergency plans	Report
Cooper	2012	Evaluating the efficacy of the AAP "pediatrics in disaster" course: the Chinese experience.	International	Disasters	Health workforce	N/A	Impact	Before-after
Evans	2016	Innovation in Graduate Education for Health Professionals in Humanitarian Emergencies.	International	Disasters	Academia	In-person training	Learning technique	Report
Silva	2021	Implementation of COVID-19 telemonitoring: repercussions in Nursing academic training.	Brazil	COVID-19	Health workforce	Virtual training	Knowledge evaluation	Report
Aghababaeian	2013	A comparative study of the effect of triage training by role-playing and educational video on the knowledge and performance of emergency medical service staffs in Iran.	Iran	Disasters	Health workforce	Blended	Knowledge evaluation	RCT
Chiu	2021	Facing the Coronavirus Pandemic: An Integrated Continuing Education Program in Taiwan.	China	COVID-19	Health workforce	Virtual training	Knowledge evaluation	Before-after
Haar	2020	Strong families: a new family skills training program for challenged and humanitarian settings: a single-arm intervention tested in Afghanistan.	Afghanistan	Disasters	Citizens and affected population	In-person training	Impact	Before-after
Shi	2020	A simulation training course for family medicine residents in China managing COVID-19.	China	COVID-19	Health workforce	Simulation	Knowledge evaluation	Before-after
Rouzier	2013	Cholera vaccination in urban Haiti.	Haiti	Cholera	Health workforce and community	In-person training	Emergency plans	Report
Abbas	2018	Peers versus professional training of basic life support in Syria: a randomized controlled trial.	Syria	Disasters	Health workforce	In-person training	Impact	RCT
Finnegan	2015	Preparing British Military nurses to deliver nursing care on deployment. An Afghanistan study.	Afghanistan	Disasters	Military	In-person training	Emergency plans	Report
Schreiber	2004	Hospital preparedness for possible nonconventional casualties: an Israeli experience.	Israel	others	Health workforce	In-person training	Emergency plans	Report
Phillips GA	2014	Capacity building for emergency care: Training the first emergency specialists in Myanmar.	Myanmar	Disasters	Health workforce	Academic training	Learning technique	Report
Sun L	2021	Intervention Effect of Time Management Training on Nurses’ Mental Health during the COVID-19 Epidemic.	China	COVID-19	Health workforce	In-person training	Impact	Before-after
Hung KKC	2021	Health Workforce Development in Health Emergency and Disaster Risk Management: The Need for Evidence-Based Recommendations.	International	Disasters	Health workforce	N/A	Emergency plans	Review
Mosquera A	2015	U.S. Public Health Service Response to the 2014–2015 Ebola Epidemic in West Africa: A Nursing Perspective.	Africa	Ebola	Health workforce	Simulation	Learning technique	Report
Bajow NA	2019	A Basic Course in Humanitarian Health Emergency and Relief: A Pilot Study from Saudi Arabia.	Saudi Arabia	Disasters	Health workforce	In-person training	Knowledge evaluation	Before-after
Chan SS	2010	Development and evaluation of an undergraduate training course for developing International Council of Nurses disaster nursing competencies in China.	China	Disasters	Health workforce	Academic training	Knowledge evaluation	Before-after
Ikeda S	2022	Introduction to the Project for Strengthening the ASEAN Regional Capacity on Disaster Health Management (ARCH Project).	Japan	Disasters	Health workforce	Academic training	Emergency plans	Report
Iskanderani AI	2021	Artificial Intelligence and Medical Internet of Things Framework for Diagnosis of Coronavirus Suspected Cases.	China	COVID-19	Health workforce	Virtual training	Learning technique	Report
Rehman H	2020	Effectiveness of basic training session regarding the awareness of Ebola virus disease among nurses of public tertiary care hospitals of Lahore.	Pakistan	Ebola	Health workforce	In-person training	Knowledge evaluation	Before-after
Díaz-Guio DA	2020	Cognitive load and performance of health care professionals in donning and doffing PPE before and after a simulation-based educational intervention and its implications during the COVID-19 pandemic for biosafety.	Colombia	COVID-19	Health workforce	Simulation	Knowledge evaluation	Before-after
Bai HX	2020	Artificial Intelligence Augmentation of Radiologist Performance in Distinguishing COVID-19 from Pneumonia of Other Origin at Chest CT.	China	COVID-19	Health workforce	Virtual training	Knowledge evaluation	Observational
Tan W;	2020	Whole-Process Emergency Training of Personal Protective Equipment Helps Healthcare Workers Against COVID-19: Design and Effect.	China	COVID-19	Health workforce	Simulation	Knowledge evaluation	Before-after
Kimani D	2022	Adopting World Health Organization Multimodal Infection Prevention and Control Strategies to Respond to COVID-19, Kenya.	Kenya	COVID-19	Health workforce	In-person training	Learning technique	Report
Hu X;	2022	Creation and application of war trauma treatment simulation software for first aid on the battlefield based on undeformed high-resolution sectional anatomical image (Chinese Visible Human dataset).	China	Disasters	Health workforce	Virtual training	Impact	Report
Bajow N	2015	Proposal for a community-based disaster management curriculum for medical school undergraduates in Saudi Arabia	Saudi Arabia	Disasters	Health workforce	Academic training	Learning technique	Report
Kesavadev J;	2021	A new interventional home care model for COVID management: Virtual Covid IP.	India	COVID-19	Health workforce	Virtual training	Learning technique	Observational
Sohn VY;	2007	From the combat medic to the forward surgical team: the Madigan model for improving trauma readiness of brigade combat teams fighting the Global War on Terror.	Iraq	War	Military	Simulation	Knowledge evaluation	Report
Cerqueira-Silva T	2021	Bridging Learning in Medicine and Citizenship During the COVID-19 Pandemic: A Telehealth-Based Case Study.	Brazil	COVID-19	Health workforce	Virtual training	Impact	Report
Barbier O	2018	Has Current French Training for Military Orthopedic Surgeons Deployed in External Operations Been Appropriately Adapted?	Africa and Afghanistan	War	Military	Academic training	Learning technique	Observational
Leochico CFD;	2021	Role of Telerehabilitation in the Rehabilitation Medicine Training Program of a COVID-19 Referral Center in a Developing Country.	Philippines	COVID-19	Health workforce	Virtual training	Learning technique	Report
Pereira BM	2010	Predeployment mass casualty and clinical trauma training for US Army forward surgical teams.	Iraq and Afghanistan	war	Military	In-person training	Impact	Report
Philip S	2022	A report on successful introduction of tele mental health training for primary care doctors during the COVID 19 pandemic.	India	COVID-19	Health workforce	Virtual training	Knowledge evaluation	Report
Brearley MB	2016	Pre-deployment Heat Acclimatization Guidelines for Disaster Responders.	Philippines	Disasters	Military	Virtual training	Knowledge evaluation	Report
Das A;	2022	Implementation of infection prevention and control practices in an upcoming COVID-19 hospital in India: An opportunity not missed.	India	COVID-19	Health workforce	Blended	Knowledge evaluation	Observational
Gareev I;	2021	The opportunities and challenges of telemedicine during COVID-19 pandemic.	International	COVID-19	Health workforce	Virtual training	Learning technique	Report
Jensen	2015	Integration of Surgical Residency Training With US Military Humanitarian Missions.	South Asia	War	Military	In-person training	Knowledge evaluation	Report
Choufani	2021	Evaluation of a fellowship abroad as part of the initial training of the French military surgeon.	Africa	War	Military	In-person training	Impact	Report
Tashkandi	2021	Nursing strategic pillars to enhance nursing preparedness and response to COVID-19 pandemic at a tertiary care hospital in Saudi Arabia	Saudi Arabia	COVID-19	Health workforce	In-person training	Knowledge evaluation	Report
Chiu	2021	Developing and Implementing a Dedicated Prone Positioning Team for Mechanically Ventilated ARDS Patients During the COVID-19 Crisis.	China	COVID-19	Health workforce	In-person training	Knowledge evaluation	Report
Operario	2016	Effect of a knowledge-based and skills-based programme for physicians on risk of sexually transmitted reinfections among high-risk patients in China: a cluster randomised trial.	China	others	Health workforce	In-person training	Knowledge evaluation	RCT
Wang	2009	Intervention to train physicians in rural China on HIV/STI knowledge and risk reduction counseling: preliminary findings.	China	others	Health workforce	In-person training	Knowledge evaluation	Before-after
El-Bahnasawy	2015	TRAINING PROGRAM FOR NURSING STAFF REGARDING VIRAL HEMORRHAGIC FEVERS IN A MILITARY HOSPITAL.	Egypt	others	Health workforce	In-person training	Impact	Before-after
Khari	2022	The Effect of E-Learning Program for COVID-19 Patient Care on the Knowledge of Nursing Students: A Quasi-Experimental Study.	Iran	COVID-19	Health workforce	Virtual training	Knowledge evaluation	Before-after
Ripoll-Gallardo	2020	Residents working with Médecins Sans Frontières: training and pilot evaluation.	Africa	Disasters	Health workforce	Blended	Impact	Report
Otu	2021	Training health workers at scale in Nigeria to fight COVID-19 using the InStrat COVID-19 tutorial app: an e-health interventional study.	Nigeria	COVID-19	Health workforce	Virtual training	Knowledge evaluation	Before-after
Lopes	2020	Adult learning and education as a tool to contain pandemics: The COVID-19 experience.	Africa	COVID-19	Citizens and affected population	In-person training	Emergency plans	Opinion
Jackson	2022	Developing and Implementing Noninvasive Ventilator Training in Haiti during the COVID-19 Pandemic.	Haiti	COVID-19	Health workforce	In-person training	Knowledge evaluation	Before-after
Sharma	2021	Effectiveness of Video-Based Online Training for Health Care Workers to Prevent COVID-19 Infection: An Experience at a Tertiary Care Level Institute, Uttarakhand, India.	India	COVID-19	Health workforce	Virtual training	Knowledge evaluation	Before-after
Daniel	2020	Responding to Palliative Care Training Needs in the Coronavirus Disease 2019 Era: The Context and Process of Developing and Disseminating Training Resources and Guidance for Low- and Middle-Income Countries from Kerala, South India.	India	COVID-19	Health workforce	Virtual training	Emergency plans	Report
Downie	2022	Remote Consulting in Primary Health Care in Low- and Middle-Income Countries: Feasibility Study of an Online Training Program to Support Care Delivery During the COVID-19 Pandemic.	Tanzania	COVID-19	Health workforce	Virtual training	Knowledge evaluation	Report
Scott	2020	Training the Addiction Treatment Workforce in HIV Endemic Regions: An Overview of the South Africa HIV Addiction Technology Transfer Center Initiative.	South Africa	others	Experts and volunteers	In-person training	Knowledge evaluation	Before-after
Usami	2018	Addressing challenges in children’s mental health in disaster-affected areas in Japan and the Philippines—highlights of the training program by the National Center for Global Health and Medicine.	Japan	Earthquake	Health workforce	In-person training	Impact	Report
Buyego	2021	Feasibility of Virtual Reality based Training for Optimising COVID-19 Case Handling in Uganda.	Uganda	COVID-19	Health workforce	Virtual training	Impact	Report
Babu	2021	Simulated Patient Environment: A Training Tool for Healthcare Professionals in COVID-19 Era.	India	COVID-19	Health workforce	Simulation	Knowledge evaluation	Before-after
Liu	2022	Development and Evaluation of Innovative and Practical Table-top Exercises Based on a Real Mass-Casualty Incident.	China	Disasters	Health workforce	Virtual training	Impact	Before-after
Fuenfer	2009	The U.S. military wartime pediatric trauma mission: how surgeons and pediatricians are adapting the system to address the need.	Afganistan and Iraq	War	Health workforce	Virtual training	Emergency plans	Report
Macht	2022	COVID-19: Development and implementation of a video-conference-based educational concept to improve the hygiene skills of health and nursing professionals in the Republic of Kosovo.	Kosovo	COVID-19	Health workforce	Virtual training	Knowledge evaluation	Report
Jobson	2019	Targeted mentoring for human immunodeficiency virus programme support in South Africa	South Africa	others	Health workforce	In-person training	Impact	Report
Zelnick	2018	Training social workers to enhance patient-centered care for drug-resistant TB-HIV in South Africa.	South Africa	others	Health workforce	In-person training	Knowledge evaluation	Report
Cena-Navarro	2022	Biosafety Capacity Building During the COVID-19 Pandemic: Results, Insights, and Lessons Learned from an Online Approach in the Philippines.	Philippines	COVID-19	Health workforce	Virtual training	Learning technique	Report
Richard	2009	Essential trauma management training: addressing service delivery needs in active conflict zones in eastern Myanmar.	Myanmar	War	Health workforce	In-person training	Knowledge evaluation	Report
Galagan	2017	Improving Tuberculosis (TB) and Human Immunodeficiency Virus (HIV) Treatment Monitoring in South Africa: Evaluation of an Advanced TB/HIV Course for Healthcare Workers.	South Africa	others	Health workforce	In-person training	Knowledge evaluation	Before-after
Irizarry	2012	Advanced Medical Technology Capacity Building and the Medical Mentoring Event: A Unique Application of SOF Counterinsurgency Medical Engagement Strategies.	Afghanistan	War	Health workforce and community	Simulation	Emergency plans	Report
Wanjiku	2022	Feasibility of project ECHO telementoring to build capacity among non-specialist emergency care providers.	Kenya	Disasters	Health workforce	Virtual training	Emergency plans	Report
Arnold	2020	Bridging the Gap Between Emergency Response and Health Systems Strengthening: The Role of Improvement Teams in Integrating Zika Counseling in Family Planning Services in Honduras.	Honduras	others	Health workforce	In-person training	Emergency plans	Before-after
Martinez	2018	Tourniquet Training Program Assessed by a New Performance Score.	Africa	War	Health workforce	In-person training	Knowledge evaluation	RCT
Kulshreshtha P	2022	Preparedness of Undergraduate Medical Students to Combat COVID-19: A Tertiary Care Experience on the Effectiveness and Efficiency of a Training Program and Future Prospects.	India	COVID-19	Health workforce	Simulation	Knowledge evaluation	Before-after
Ahluwalia	2021	Effectiveness of remote practical boards and telesimulation for the evaluation of emergency medicine trainees in India.	India	Disasters	Health workforce	Virtual training	Impact	Report
Al-Hadidi	2021	Homemade cardiac and vein cannulation ultrasound phantoms for trauma management training in resource-limited settings.	Syria	Disasters	Health workforce	Simulation	Impact	Report
Patel	2020	"Emerging Technologies and Medical Countermeasures to Chemical, Biological, Radiological, and Nuclear (CBRN) Agents in East Ukraine"	Ukraine	War	Health workforce	In-person training	Emergency plans	Report
Wang	2022	Practical COVID-19 Prevention Training for Obstetrics and Gynecology Residents Based on the Conceive-Design-Implement-Operate Framework.	China	COVID-19	Health workforce	Academic training	Impact	RCT
de Lesquen	2020	Adding the Capacity for an Intensive Care Unit Dedicated to COVID 19, Preserving the Operational Capability of a French Golden Hour Offset Surgical Team in Sahel.	Niger and Mali	COVID-19	Military	Academic training	Emergency plans	Report
Ye	2021	Point-of-care training program on COVID-19 infection prevention and control for pediatric healthcare workers: a multicenter, cross-sectional questionnaire survey in Shanghai, China.	China	COVID-19	Health workforce	Virtual training	Impact	Before-after
Bhattacharya	2020	Impact of a training program on disaster preparedness among paramedic students of a tertiary care hospital of North India: A single-group, before-after intervention study.	India	Disasters	Health workforce	Blended	Impact	Before-after
Wong	2022	ECMO simulation training during a worldwide pandemic: The role of ECMO telesimulation.	China	COVID-19	Health workforce	Virtual simulation	Impact	Before-after
Fernández-Miranda	2021	Developing a Training Web Application for Improving the COVID-19 Diagnostic Accuracy on Chest X-ray.	Chile	COVID-19	Health workforce	Virtual training	Knowledge evaluation	Report
Khoshnudi	2022	Comparison of the effect of bioterrorism education through two methods of lecture and booklet on the knowledge and attitude of nurses of Shams Al-Shomus Nezaja Hospital.	Iran	Disasters	Health workforce	In-person training	Knowledge evaluation	Before-after
Liesveld	2022	Teaching disaster preparedness to pre-licensure students: A collaborative project during the pandemic.	International	COVID-19	Health workforce	Virtual training	Impact	Report
Shilkofski	2017	Pediatric Emergency Care in Disaster-Affected Areas: A Firsthand Perspective after Typhoons Bopha and Haiyan in the Philippines.	Philippines	Disasters	Health workforce	N/A	Emergency plans	Review
Bankole	2021	KNOWLEDGE OF HEALTH WORKERS ON CHOLERA MANAGEMENT IN OYO STATE: RESULTS OF A TRAINING INTERVENTION.	Nigeria	Cholera	Health workforce	In-person training	Knowledge evaluation	Before-after
	2023	The Use of Open-Source Online Course Content for Training in Public Health Emergencies: Mixed Methods Case Study of a COVID-19 Course Series for Health Professionals	International	COVID-19	Health workforce	Virtual training	Learning technique	Qualitative
Klomp	2020	CDC’s Multiple Approaches to Safeguard the Health, Safety, and Resilience of Ebola Responders	Africa	Ebola	Health workforce	In-person training	Emergency plans	Report
Van Hulle	2020	Tailoring malaria routine activities within the covid-19 pandemic: A risk and mitigation assessment of eight countries in West Africa	West Africa	others	Health workforce and community	Virtual training	Emergency plans	Report
Guerrero-Torres	2020	Impact of Training Residents to Improve HIV Screening in a Teaching Hospital in Mexico City	Mexico	others	Health workforce	In-person training	Knowledge evaluation	Before-after
Sena	2020	Disaster preparedness training in emergency medicine residents using a tabletop exercise	International	Disasters	Health workforce	In-person training	Learning technique	Before-after
Mitchell	2020	A partnership to develop disaster simulations for nursing students in response to climate change: description of a programme in Bluefields, Nicaragua, and Virginia, USA	Nicaragua y USA	Disasters	Health workforce	In-person training	Learning technique	Report
Amini	2019	Epidemiological profile of crimean congo hemorrhagic fever in afghanistan: A teaching-case study	Africa	others	Health workforce	In-person training	Learning technique	Report
Al-Mayahi	2019	Surveillance gaps analysis and impact of the late detection of the first middle east respiratory syndrome case in south batinah, oman: A teaching case-study	Africa	others	Health workforce	In-person training	Learning technique	Report
Pelican	2019	Building an Ebola-Ready workforce: Lessons learned on strengthening the global workforce through university networks	International	Ebola	Health workforce	In-person training	Emergency plans	Report
Peters	2019	Development and pilot testing of an infection prevention and control (IPC) tool for humanitarian response to outbreaks and natural disasters	Zambia	others	Health workforce	In-person training	Emergency plans	Before-after
Keita	2018	Impact of infection prevention and control training on health facilities during the Ebola virus disease outbreak in Guinea	West Africa	Ebola	Health workforce	In-person training	Knowledge evaluation	Observational
Edem-Hotah	2018	Utilizing Nurses to Staff an Ebola Vaccine Clinical Trial in Sierra Leone during the Ebola Outbreak	Sierra Leone	Ebola	Health workforce	In-person training	Emergency plans	Report
Mbanjumucyo	2018	Major incident simulation in Rwanda: A report of two exercises	Rwanda	Disasters	Health workforce	Simulation	Emergency plans	Report
Bruning M.D	2018	The kid next door-raising awareness of Civilian health care providers to the needs of military children-a tactical approach to education	Iraq and Afganistan	War	Health workforce	In-person training	Knowledge evaluation	Before-after
Andrews R	2018	Computer-assisted disaster response: Benefits for global healthcare	Africa	Disasters	Health workforce	In-person training	Emergency plans	Report
Githuku	2017	Cholera outbreak in homa bay county, kenya, 2015	Africa	Cholera	Health workforce	In-person training	Learning technique	Report
Jones-Konneh	2017	Intensive education of health care workers improves the outcome of ebola virus disease: Lessons learned from the 2014 outbreak in Sierra Leone	Sierra Leone	Ebola	Health workforce	In-person training- simulation	Impact	Report
Phaup	2017	Increasing access to HIV treatment and care services for key populations in zambia: A partnership approach to strengthening local capacity to provide sensitivity training to health workers	Zambia	others	Health workforce	In-person training	Emergency plans	Report
Nwandu	2017	Sustainable pepfar funded in service HIV training delivery models: A training impact evaluation from nigeria	Nigeria	others	Health workforce	In-person training	Knowledge evaluation	Before-after
Laudisoit	2017	A One Health team to improve Monkeypox virus outbreak response: An example from the Democratic Republic of the Congo	Democratic Republic of Congo	others	Health workforce	In-person training	Emergency plans	Report
Umar	2017	Learningthroughservice: ’shifa homes’a project of Shifa College of Medicine for rehabilitation of flood victims	Pakistan	Disasters	Citizens and affected population	In-person training	Emergency plans	Report
Vaz	2016	The role of the polio program infrastructure in response to Ebola virus disease outbreak in Nigeria 2014	Nigeria	Ebola	National Institutions	In-person training	Emergency plans	Report
Houben	2016	TIME Impact—a new user-friendly tuberculosis (TB) model to inform TB policy decisions	International	others	National Institutions	Virtual training	Emergency plans	Report
Umoren	2016	From global to local: Virtual environments for global-public health education	International	Disasters	Citizens and affected population	Virtual training	Emergency plans	Report
Garde	2016	Implementation of the first dedicated Ebola screening and isolation for maternity patients in Sierra Leone	Sierra Leone	Ebola	Health workforce	In-person training	Impact	Report
Toda	2016	The impact of a SMS-based disease outbreak alert system (mSOS) in Kenya	Kenya	others	Health workforce	In-person training	Impact	RCT
Shao X	2015	Evaluation of anti-Ebola training system in the Medical Team to Liberia and some suggestion	China	Ebola	Military	Blended	Emergency plans	Report
Ali	2015	Applicability of the advanced disaster medical response (ADMR) course, Trinidad and Tobago	Trinidad y Tobago	Disasters	Health workforce	In-person training	Learning technique	Before-after
Livingston	2015	Healthcare capacity building in Haiti: Training healthcare and non-healthcare providers in basic cardiopulmonary resuscitation	Haiti	Disasters	Health workforce and community	Simulation	Learning technique	Report
Berry	2015	How to set up an Ebola isolation unit: Lessons learned from Rokupa	Sierra Leone	Ebola	Health workforce	In-person training	Impact	Report
El-Bahnasawy	2015	Mosquito borne West Nile virus infection as a major threat	Egypt	others	Health workforce	In-person training	Impact	Report
Ariel	2014	The birth of family therapists: The kosova systemic family therapy training program	Kosovo	War	Health workforce	In-person training	Learning technique	Report
Reynolds	2014	Training health workers for enhanced monkeypox surveillance, Democratic Republic of the Congo	Congo	others	Health workforce	In-person training	Impact	Before-after
Acevedo	2013	Organization of the health system response to the 2009 H1N1 influenza pandemic in a hospital in Lima, Peru	Peru	others	Health workforce	In-person training	Emergency plans	Report
Hasanovic	2013	EMDR training for bosnia-herzegovina mental health workers in sarajevo as continuity of the building of psychotherapy capacity aftermath the 1992–1995 war	Bosnia-herzegovina	War	Health workforce	virtual training	Impact	Report
Hasanovic	2013	Training of bosnia-herzegovina mental health professionals in group analysis as the factor of development of culture of dialogue in the aftermath of the 1992–1995 war	Bosnia-herzegovina	War	Health workforce	In-person training	Impact	Report
Diaz	2013	Development of a severe influenza critical care curriculum and training materials for resource-limited settings	International	others	Health workforce	In-person training	Learning technique	Report
Asgary	2013	Comprehensive on-site medical and public health training for local medical practitioners in a refugee setting	Africa	Disasters	Health workforce	In-person training	Knowledge evaluation	Before-after
Plani	2012	Development of a hospital disaster plan and training exercises for chris hani baragwanath academic hospital and resource-limited countries	South Africa	Disasters	Health workforce	In-person training	Learning technique	Report
Wurapa	2012	Establishing a tropical medicine training program for the us department of defense (DOD) in kintampo, ghana: Overcoming challenges	Ghana	Disasters	Health workforce	In-person training	Emergency plans	Report
Chihanga	2012	Toward malaria elimination in Botswana: A pilot study to improve malaria diagnosis and surveillance using mobile technology	Botswana	others	Health workforce	virtual training	Learning technique	Report
Garnett	2012	Using south-south collaboration to strengthen midwifery skills and competencies in South Sudan	South Sudan	War	Health workforce	In-person training	Emergency plans	Report
Grosso	2012	The role of the international anaesthetist in the professional training and management of the anaesthesia nurses. 12 years of experience of emergency Italian NGO in Afghanistan	Afghanistan	War	Health workforce	In-person training	Emergency plans	Report
Norton	2012	The power of immersion; Training health personnel for disaster humanitarian responses	International	Disasters	Health workforce	In-person training	Emergency plans	Report
He	2011	The urgent rehabilitation technique education program for Wenchuan earthquake victims	China	Earthquake	Health workforce	In-person training	Emergency plans	Report
Sadiwa	2011	Addressing developmental delays among African children in post-conflict areas: An E-health approach	sierra Leone	War	Experts and volunteers	virtual training	Learning technique	Report
Oliveira	2011	Esperience in confronting the H1N1 epidemy	Brasil	others	Health workforce	In-person training	Impact	Report
Mbabazi	2011	Phase 1 implementation of male circumcision as a comprehensive package of HIV prevention in Rwanda	Rwanda	others	Health workforce	In-person training	Emergency plans	Report
Darby	2011	Multi-modal training for adult ICU nurses caring for paediatric patients in a war zone	Afghanistan	War	Health workforce	In-person training	Knowledge evaluation	Report
Way	2011	A modality of disaster response: Cyclone nargis and psychological first aid	Burma	Disasters	Health workforce and community	In-person training	Emergency plans	Report
Hasanovic	2011	Emdr training for mental health therapists in postwar bosniaherzegovina who work with psycho-traumatized population for increasing their psychotherapy capacities	Bosnia-herzegovina	War	Health workforce	In-person training	Emergency plans	Report
Oleribe	2010	From strategy to action: The vital roles of trained field epidemiologists and laboratory management professionals in epidemic control and prevention in Tanzania	Tanzania	others	Health workforce	In-person training	Emergency plans	Report
Kuhls	2009	International disaster training: Advanced disaster life support (ADLS) improves Thai physician and nurse confidence to respond to mass casualty disasters	Thailand	Disasters	Health workforce	In-person training	Knowledge evaluation	Before-after
van der Walt	2006	The effect of a CPD training (educational) intervention on the level of HIV knowledge of pharmacists	South Africa	others	Health workforce	virtual training	Knowledge evaluation	RCT
Peltzer	2006	A controlled study of an HIV/AIDS/STI/TB intervention with traditional healers in KwaZulu-Natal, South Africa	South Africa	others	Health workforce	In-person training	Knowledge evaluation	RCT
Wondmikun	2005	Successful coupling of community attachment of health science students with relief work for drought victims	Ethiopia	Disasters	Health workforce	In-person training	Impact	Report
Kabir	2021	Association between preference and e-learning readiness among the Bangladeshi female nursing students in the COVID-19 pandemic: a cross-sectional study	Bangladesh	COVID-19	Health workforce	Virtual training	Learning technique	Cross-sectional
AlOsta	2023	Jordanian nursing students’ engagement and satisfaction with e-learning during COVID-19 pandemic	Jordania	COVID-19	Health workforce	Virtual training	Learning technique	Report
Severini	2023	How to incorporate telemedicine in medical residency: A Brazilian experience in pediatric emergency	Brasil	COVID-19	Health workforce	in-person training	Learning technique	Before-after
Conyers	2023	Where There’s a War, There’s a Way: A Brief Report on Tactical Combat Casualty Care Training in a Multinational Environment	International	War	Military	In-person training	Emergency plans	Report
Farhat	2022	The educational outcomes of an online pilot workshop in emergencies	Middle-east	Disasters	Health workforce	Virtual training	Emergency plans	Before-after
Mitchell	2023	Multimodal learning for emergency department triage implementation: experiences from Papua New Guinea during the COVID-19 pandemic	Papua New Guinea	Disasters	Health workforce	Virtual training	Knowledge evaluation	Before-after
Wang	2022	Rapid virtual training and field deployment for COVID-19 surveillance officers: experiences from Ethiopia	Ethiopia	COVID-19	Health workforce	Virtual training	Emergency plans	Report
Suresh	2021	Predeployment training of Army medics assigned to prehospital settings	International	War	Health workforce	N/A	Knowledge evaluation	Report
Popova	2022	EXPERIENCE IN ORGANIZING URGENT TRAINING FOR GI PROFESSIONALS ON EMERGENCY CARE FOR ABDOMINAL INJURIES DURING THE WAR IN UKRAINE	Ukraine	War	Health workforce	Virtual training	Emergency plans	Report
Beckmann	2022	Training of psychotherapists in post-conflict regions: A Community case study in the Kurdistan Region of Iraq	Iraq	War	Health workforce	In-person training	Emergency plans	Report
Susanti	2022	The Effect of Caring Training on the Implementation of Caring Behavior and Work Culture of Nurses in Providing Services to COVID-19 Patients in an Indonesia’s National Referral Hospital	Indonesia	COVID-19	Health workforce	In-person training	Impact	Before-after
Canavese	2022	Massive Open Online Courses as Strategies to Address Violence through the Training of Health and the Intersectoral Professionals in Brazil	Brasil	COVID-19	Health workforce	Virtual training	Learning technique	Report
Han	2022	Effect Analysis of "Four-Step" Training and Assessment Tool in the Prevention and Control of COVID-19	China	COVID-19	Health workforce	Virtual training	Learning technique	Report
Wood	2022	Evaluation of virtual online delivery of United Nations Office on Drugs and Crime (UNODC) national training on novel psychoactive substances (NPS) to healthcare professionals in Mauritius and the Seychelles during the COVID-19 pandemic	Mauritius and the Seychelles	COVID-19	Health workforce	Virtual training	Knowledge evaluation	Before-after
Kamal	2022	Virtual Training on IGRT: A Unique Initiative of a Private Cancer Center from a Developing Country with Regional Academic Collaboration during COVID-19 Pandemic	Bangladesh	COVID-19	Health workforce	Virtual training	Knowledge evaluation	Report
Fernandes Canesin	2022	Use of an innovative humanized virtual digital interactive heart failure clinical cases training strategy for cardiologist in the covid 19 pandemic	Brazil- Portugal and US	COVID-19	Health workforce	Virtual training	Learning technique	Observational
Toro	2022	A Simulated Hospital in a COVID-19 Pandemic Environment for Undergraduate Neurology Students	Colombia	COVID-19	Health workforce	simulation	Knowledge evaluation	Observational
Wong	2022	Better Surgical Ward Round: Replicating Near-Peer Teaching (NPT) on a virtual international platform during the COVID-19 pandemic.	International	COVID-19	Health workforce	Virtual training	Learning technique	Before-after
Ordonez Juarez	2022	Virtual Learning Environment for Surgery Residents in a Third Level Hospital at Mexico City, a Teaching Alternative	Mexico	COVID-19	Health workforce	Virtual training	Knowledge evaluation	Report
Payne	2022	The Development and Evaluation of Online Home Palliative Training During COVID-19 Pandemic in South Africa	South Africa	COVID-19	Health workforce	Virtual training	Impact	Report
Kumar	2022	Effectiveness of virtual versus inperson training of the FCCS course: A comparative study	Ghana, Nigeria, Augusta	COVID-19	Health workforce	Virtual training	Knowledge evaluation	Observational
Kalayasiri	2021	Training of psychiatry and mental health in a low- and middle-income country: Experience from Thailand before and after COVID-19 outbreak	Thailand	COVID-19	Health workforce	Virtual training	Emergency plans	Report
Tang	2021	Combat casualty care training of Chinese peacekeeping military doctors: An evaluation of effectiveness	China	War	Military	In-person training	Knowledge evaluation	Report
Siddiqui	2021	The impact of a "one day basic intensive care training program" on knowledge of non-intensivists during the COVID-19 pandemic	India	COVID-19	Health workforce	In-person training	Knowledge evaluation	Before-after
Groninger	2021	Project ECHO palliative care: Impact of TELE-mentoring and teaching for healthcare providers working with rohingya refugees in Bangladesh	Bangladesh	War	Health workforce	Virtual training	Emergency plans	Report
Daniel	2021	Evaluation of the faculty experience in developing and delivering palliative care e-resource toolkit for COVID-19 for low and middle income countries (LMICS)	international	COVID-19	Health workforce	Virtual training	Emergency plans	Before-after
Kharel	2021	Impact of a virtual COVID-19 trainer of trainers program implemented via an academic-humanitarian collaboration	international	COVID-19	Health workforce	Virtual training	Learning technique	Before-after
Thakre	2020	Evaluation of effectiveness of Covid-19 training and assessment of anxiety among nurses of a tertiary health care center during the Corona Virus pandemic-an experimental study	India	COVID-19	Health workforce	In-person training	Emergency plans	Report
Rishipathak	2021	Assessing the effectiveness of online teaching methodology among emergency medical professionals in Pune, India	India	COVID-19	Health workforce	Virtual training	Knowledge evaluation	Report
Khoja	2016	Impact of simple conventional and Telehealth solutions on improving mental health in Afghanistan	Afghanistan	COVID-19	Health workforce	Virtual training	Emergency plans	Report
Bernstein	2022	The Power of Connections: AAP COVID-19 ECHO Accelerates Responses During a Public Health Emergency	USA	COVID-19	Health workforce	Virtual training	Learning technique	Report
Hunt	2022	Facilitating Real-Time, Multidirectional Learning for Clinicians in a Low-Evidence Pandemic Response	USA	COVID-19	Health workforce	Virtual training	Impact	Report
Hunt	2021	Virtual Peer-to-Peer Learning to Enhance and Accelerate the Health System Response to COVID-19: The HHS ASPR Project ECHO COVID-19 Clinical Rounds Initiative	USA	COVID-19	Health workforce	Virtual training	Impact	Report
Lingum	2021	Building Long-Term Care Staff Capacity During COVID-19 Through Just-in-Time Learning: Evaluation of a Modified ECHO Model	Canada	COVID-19	Health workforce	Virtual training	Impact	Report
Stephens	2022	Adapting a Telehealth Network for Emergency COVID-19 Pandemic Response, 2020–2021	International	COVID-19	Health workforce	Virtual training	Impact	Report
Begay	2021	Strengthening Digital Health Technology Capacity in Navajo Communities to Help Counter the COVID-19 Pandemic	USA	COVID-19	Health workforce	Virtual training	Emergency plans	Report

### Overview of study characteristics

Most of the articles were descriptive studies on experiences in different countries [13–177], followed by before-after studies [170, 174, 178–261]. Also, we found 19 randomized controlled trials [262–280], 14 cross-sectional studies [281–294], 12 observational studies [295–306], 7 reviews [307–313], 5 qualitative studies [314–318] and 3 opinion pieces [319–321]. (S2 Table in S1 File)

### Emergency context

**Fig 2:**
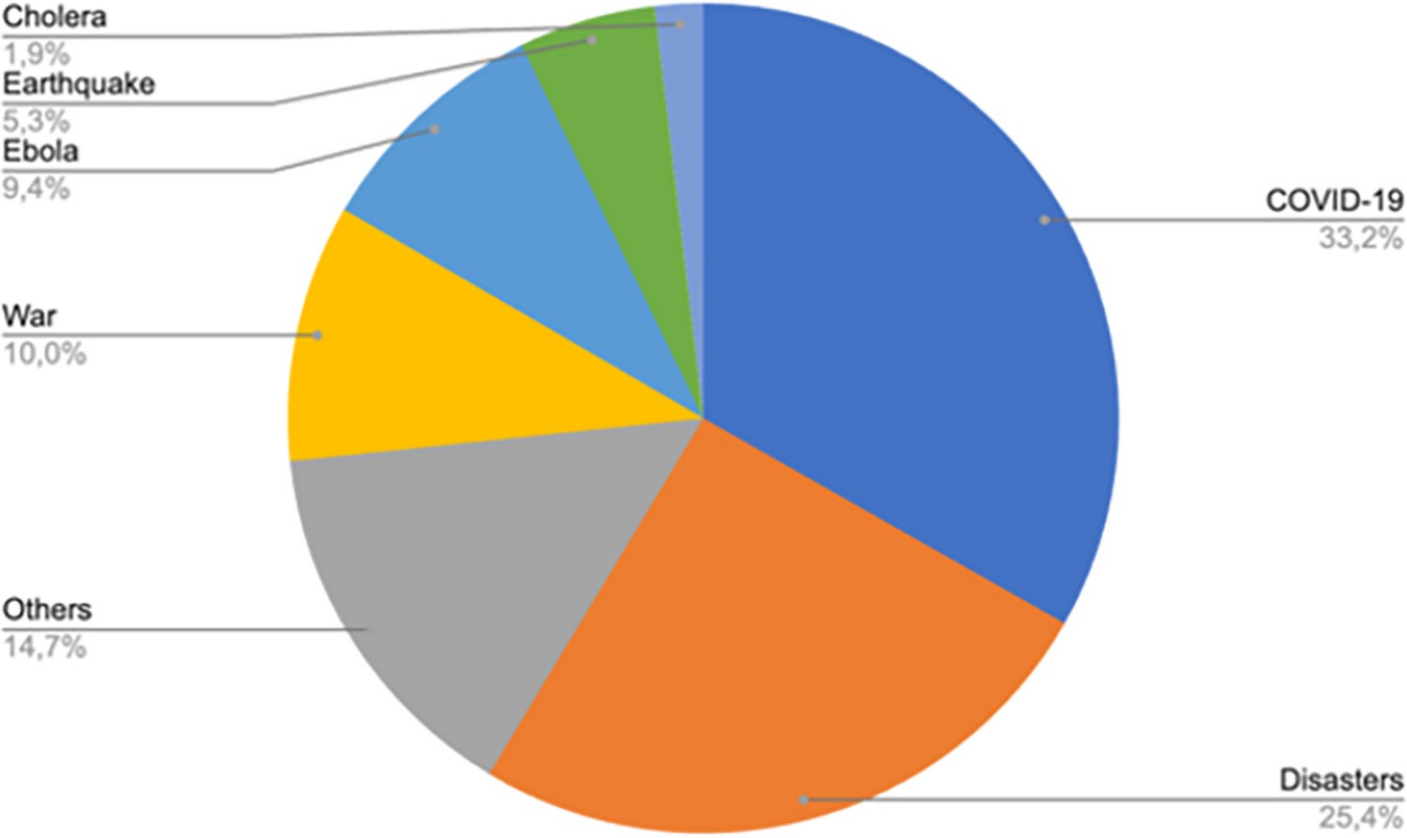
Type of emergency distribution.

COVID-19, disasters in general, Ebola and wars were the most frequent topics. This is related to the publication dates because most studies have been published during these health emergencies (Ebola, 2015 and COVID-19 during 2020, 2021 and 2022) (Fig 2)

### Location

Evidence was found in 67 countries and regions: most studies were conducted in China (n = 36), mainly evidence about COVID-19 and earthquakes, India (n = 19) and South Africa (n = 15). A large number of the learning evidence was also derived from several countries in Africa (n = 25) and also international settings (n = 23).

### Interventions, learning methods and tools

Different types of learning interventions are described across the studies. The majority of the studies used in-person training as an intervention [13, 15, 19–23, 31, 34, 35, 38–43, 47, 49–51, 53, 56, 62, 67, 68, 71, 73, 74, 76–79, 81–83, 88, 94, 98–101, 105, 109, 110, 112, 119, 121, 123–127, 129–132, 134, 136, 137, 139–142, 144, 145, 147–152, 154–160, 162, 166, 173, 175, 179–183, 185, 186, 188–190, 194, 196, 198, 199, 201, 207, 211, 213, 215–218, 221, 223, 224, 226, 229, 230, 233, 235, 238, 239, 244–246, 248–255, 258, 260, 262, 263, 265, 267, 269, 270, 272, 274, 276, 278, 280, 283, 289–291, 293, 295, 296, 299, 303, 314, 316, 318, 319, 321, 322], followed by virtual training.

Lectures, discussions, role-playing, ’hands-on’ basic skills training, materials, videos and simulations were used as in-person modalities. Additionally, 9 studies [27, 84, 86, 90, 118, 225, 264, 298, 308] suggested a professional approach (such as Masters degrees and postgraduate studies in universities) as a way of preparing healthcare workers for health emergencies.

Within the virtual modalities, studies describe the use of telemedicine [92, 93, 95, 97, 193, 284, 290], Massive Online Open Courses (MOOC) [29, 167, 209, 281, 297], social Media [16, 266, 282, 285], gamification [184, 275], virtual simulation [89, 273], artificial intelligence [87, 301] and mobile devices [148, 203].

**Fig 3:**
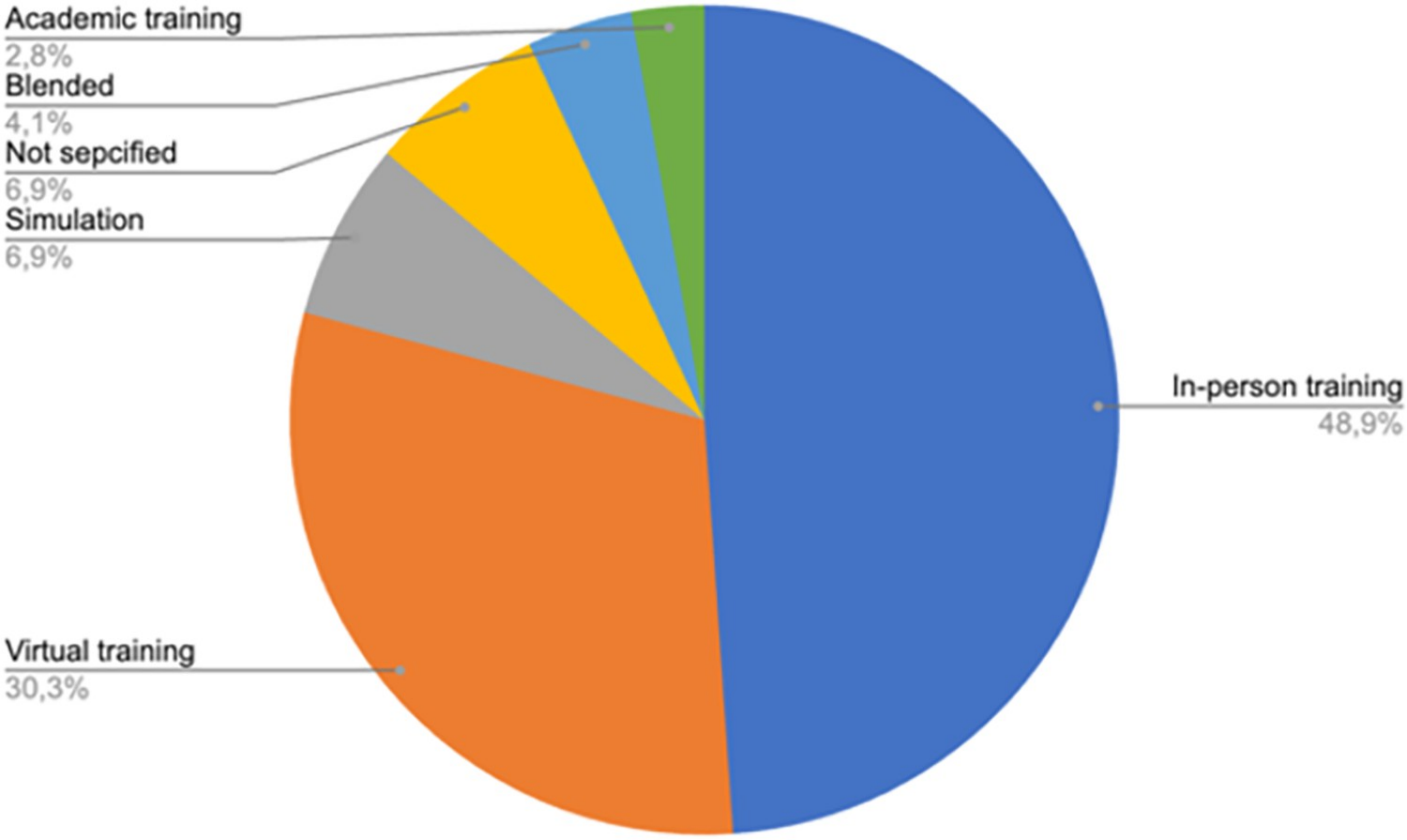
Type of learning method distribution.

A few studies used blended format, usually workshops followed by virtual training (in-person and virtual) [36, 45, 55, 64, 65, 102, 127, 128, 153, 192, 242, 271, 302, 319–321] (Fig 3)

### Learners

Most of the learning interventions were directed towards the health workforce such as medical doctors, nurses, dentists, medical students, laboratory staff and paramedical students. Some learning interventions were also aimed at military personnel, citizens and affected population, volunteers, academia and national institutions. (S3 Table in S1 File)

### Learning content

Curricula content was mostly focused on disease and disaster management. Some content was about personal safety and security [49, 50, 55, 204–206, 208, 209], waste management [66, 212, 299], mental health [95, 105, 166, 172, 177, 213, 221, 235, 259, 277, 293], infection control and prevention [139, 168, 241, 264, 302], pest control [65, 66, 70, 212, 216, 299], triage [16, 62, 91, 94, 207, 271, 290] and stress management [31, 182, 270]. A smaller number of curricula included surgical training [98, 99, 165, 323], time management [223], stigma [269], language and local culture [20], humanitarian law and leadership [102], chemical incidents [214] and dealing with ethical dilemmas [72].

## Prominent topic areas

Four prominent topic areas were identified:

1. Knowledge acquisition: Articles that analyzed how much knowledge was acquired during learning interventions as a way to assess effectiveness of the intervention methods.
2. Emergency plans: Learning recommendations focusing on how governments and institutions should be prepared to face health emergencies.
3. Impact of the learning intervention: Articles that analyzed how learning and training during health emergencies had an impact on trainees and affected populations.
4. Training method: Articles that focus on different learning methods and tools that can be used for knowledge transfer during a health emergency.

**Fig 4:**
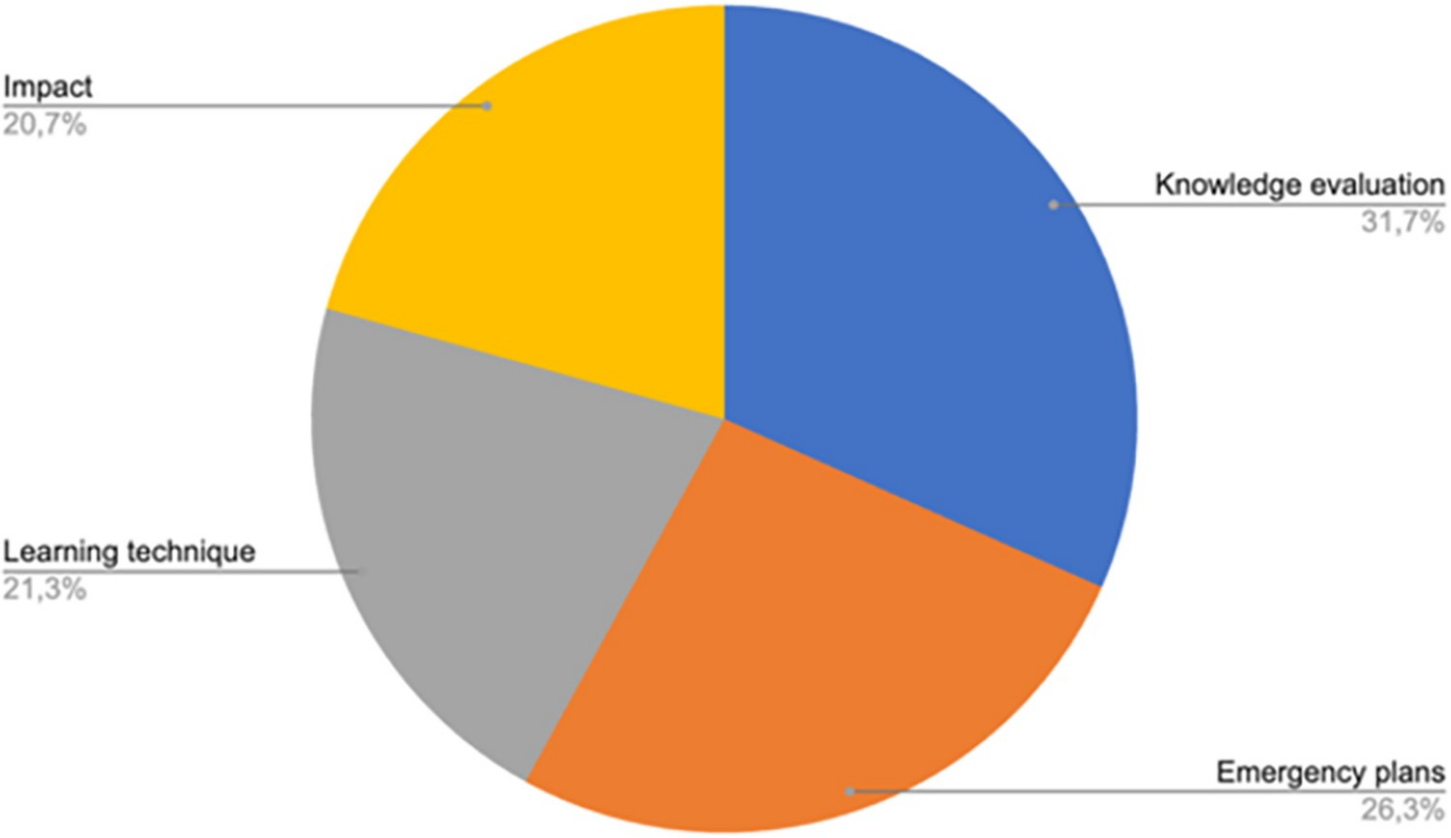
Prominent topic areas distribution.

The distribution of the prominent topic areas across the evidence is shown in Fig 4.

The largest number of articles found (n = 101) correspond to “Knowledge Evaluation”, followed by articles corresponding to “Emergency Plans” (n = 84) and “Training Method” (n = 68). Finally, “Impact” was analyzed in 66 studies.

### Knowledge acquisition

For knowledge evaluation, 101 studies [18, 19, 34, 42, 43, 49, 66, 80, 91, 95, 96, 98, 100, 101, 104, 108, 110, 112, 117, 156, 164, 169, 170, 173, 176, 178–181, 183, 186–188, 190, 195–199, 201, 203, 206–208, 212, 213, 215, 217, 218, 220, 222, 224–229, 231–236, 238, 240, 244–246, 249, 250, 253, 254, 257, 259, 260, 265–267, 269, 271, 273, 274, 279, 280, 284, 285, 287, 289–291, 293, 295, 299, 301–303, 305, 306, 316, 318] provided information about how a learning intervention during a health emergency can impact the knowledge of trainees. According to the results of these studies, learning interventions were helpful for improving knowledge, preparedness and confidence of trainees. Also, improved knowledge had a positive impact on diagnosis, and health outcomes such as rate of infected patients.

### Emergency plans

84 studies [26, 36, 38–40, 44, 50–52, 54, 55, 57, 60, 61, 63, 64, 67, 69, 71, 73–75, 78, 81–83, 86, 103, 107, 113, 114, 118, 119, 121, 122, 126–129, 131–135, 137, 142, 147, 149, 150, 152, 155, 157–159, 162, 163, 165, 166, 172, 174, 175, 177, 184, 191, 239, 248, 256, 263, 268, 275, 286, 288, 307–312, 317, 319, 320, 324–326] described the use of learning interventions as part of their emergency plans from countries and health institutions: 65 are reports, 6 are reviews and the rest of the evidence are randomized control trials, before and after studies, opinions, cross-sectional and qualitative studies. The evidence described how countries and hospitals should train healthcare workers, military, citizens and students in environmental emergencies (earthquake, tsunami, typhoons and disasters in general), biological emergencies (COVID-19, HIV, syphilis, Ebola, Cholera and Zika) and armed conflicts (war and chemical emergencies). The objective of this training was to prepare different populations for a future emergency and this explains why these interventions were not offered during an emergency.

As for the randomized controlled trials (RCT), Ma et al [275] compared the use of a gaming technique versus a disaster simulation and Lee et al [268] compared two video techniques: basic response to a fire versus generic volcanic emergency. In both studies, knowledge competence and response were statistically higher in the intervention groups (gaming technique and video response to fire emergencies).

### Training methods

On training methods, 68 studies [13–17, 20–25, 27–30, 32, 33, 37, 41, 45, 47, 48, 53, 58, 59, 62, 65, 68, 79, 84, 85, 87, 88, 90, 93, 97, 111, 123–125, 138, 141, 145, 146, 148, 153, 161, 167, 168, 170, 193, 204, 214, 216, 247, 251, 255, 261, 294, 297, 298, 300, 304, 313, 321, 322, 327] focused on describing different training methods that can be used during a health emergency. 15 studies described in-person training methods, and the rest of them used a virtual training method such as simulation, telemedicine, MOOC, videos or artificial intelligence.

MOOCs were used for cholera [29] and COVID-19 [167, 297, 315], and all of these studies showed that this method can be useful to disseminate trustworthy information in low- and middle-income countries and fill the gap of information during health emergencies.

Artificial intelligence [87] was used during COVID-19 to help radiologists to efficiently and timely diagnose suspected COVID-19 patients.

Videos [204] were implemented in Burkina Faso during a dengue epidemic. This study concluded that while videos are effective for knowledge transfer and training health professionals, the narrative genre of the videos can influence knowledge acquisition.

### Impact of learning interventions

Two studies measured impact as satisfaction in citizens and affected populations: One of them [262] trained parents of high-risk children about malaria prevention using an in-person technique. Participant satisfaction was assessed using a qualitative approach, however the main challenge reported was adherence to the course. The other study [185] reported on in-person training of Syrian refugee mothers of children with autism about overcoming trauma caused by war. Satisfaction was analyzed and improvements were recommended by the attendees, such as using online methods during the course.

In the rest of the evidence that reported satisfaction as an outcome, it was measured as self-perceived, as was mental health status of healthcare workers, well-being and preparedness.

27 studies analyzed how training during a health emergency impacted on the quality of life of healthcare workers, citizens and volunteers. The endpoints included: acceptability [296], anxiety [210], confidence [194], mental health [46, 200, 221, 223, 277, 314], preparedness [136, 140, 143, 144, 154, 160, 258, 270, 278, 328], satisfaction [31, 171, 199, 329–331], community cohesion [270] and social adaptation [292]. Most of the learning interventions were given after health emergencies occurred. This related to the fact that quality of life of the workers would be affected after the occurrence of these events. Two studies [194, 210], however, analyzed how quality of life could be improved by receiving prior training.

## Discussion

We found 319 studies that analyzed different learning interventions and training methods during health emergencies. Information about virtual training (including online platforms, artificial intelligence, MOOC, the use of mobile devices, social networks and telemedicine) arose mainly during the COVID-19 pandemic. Before the pandemic, most of the studies focused on in-person training. This change could be explained by technological advancements, the need for new information that had to be updated quickly and also because of social distancing recommendations. Of note, access to new technologies was scaled up after 2020 as the pandemic accelerated this process.

Many studies analyzed the impact of training on clinical outcomes such as patient survival [20, 216], rate of worker infection [34, 42, 88], number of hospitalizations [100, 300] and number of correct diagnoses [87, 301]. These findings will be important when developing guidance since these outcomes can be useful for decision making and selection of appropriate learning interventions and methods. Finally, the importance of coordinated work between different institutions [17, 26, 29, 39, 113, 239, 286], universities, governments and non-governmental organizations was noted in several studies. How best to foster coordinated work between institutions may be of great interest for future research.

We also found some potential evidence gaps that are worth investigating in future research studies. First, there is limited evidence on the content of learning interventions, especially regarding ethical dilemmas and how to solve them [72]. Also, we found a scarcity of evidence on end-of-life management during emergencies such as end-of-life care and managing deceased people. Subsequently, we identified other evidence gaps such as lack of robust evaluation of the effect of learning transfer, as most studies rely on self-reports (also referred to as post-training ‘smile-sheets’) to evaluate transfer of learning [332–334]. These studies provide limited actionable data to determine the effectiveness of training programs, as getting a favorable reaction from learners does not guarantee that learning transfer has been achieved [335]. Further, too little research has examined the accountability of trainers for transfer in terms of using transfer-enhancing strategies, and how trainers are being evaluated [336]

In addition, most studies focused on information dissemination to build skills rather than employing evidence-informed training methods for performance improvement at the time of emergencies. Poor learning design and evaluation methods can result in learning being wasted [332]. Lastly, there was a lack of evidence on managing emergencies through teamwork, effective communication, and stress management. After all, factors such as stress, poor team dynamic due to hierarchical issues, and poor communication can lead to morbidity or mortality.

Overall, our scoping review on ‘just in time’ learning in crises situations dovetails with McGill and colleagues scoping review using the same methods to identify subsequent knowledge exchange processes in health crises [11].

## Limitations and strengths

This scoping review has potential limitations and strengths. One such limitation was the time constraint to deliver the scoping review quickly. Despite following standard scoping review processes, the search was not as robust as when performing a systematic review. However, once the *Learning in Health Emergencies* guidance is under way and new systematic reviews on the subject are commissioned, new evidence may arise to complement these findings. However, this scoping review fulfills a vital first step in establishing the amount and type of evidence from which subsequent systematic reviews were commissioned with subsequent critical and in-depth analysis. This work is the first to address this topic and will serve as a precursor for more comprehensive investigations. The scoping review followed an a priori protocol and was undertaken by a diverse team with varied expertise in the topic and scoping review methods.

## Conclusion

Research on learning and learning dissemination during health emergencies has revealed considerable advancements, particularly in virtual learning. Overall, it is evident that learning during health emergencies appears to improve knowledge, management, quality of life, satisfaction and clinical outcomes. All the information provided by this review can give decision makers tools to select different types of learning interventions and methods for healthcare workers, volunteers, military, civilians and governments. This scoping review may also be useful for future research to address the identified evidence gaps.

## Supporting information

S1 File (DOCX)
