# Correction: Learning interventions and training methods in health emergencies: A scoping review

**DOI:** 10.1371/journal.pone.0342018

**Published:** 2026-01-29

**Authors:** Heini Utunen, Giselle Balaciano, Elham Arabi, Anna Tokar, Aphaluck Bhatiasevi, Jane Noyes

The ORCID iD is missing for the first author. Please see the author’s ORCID iD here:

Author Heini Utunen’s ORCID iD is: 0000-0002-0509-5067

(https://orcid.org/0000-0002-0509-5067).
