# Supplementary material for: Learning interventions and training methods in health emergencies: A scoping review
Source: PLoS One. 2024 Jul 16;19(7):e0290208. doi: 10.1371/journal.pone.0290208 (PMC11251632; doi:10.1371/journal.pone.0290208)
Supplement: S1 File — (DOCX) [file pone.0290208.s001.docx]

Table 1. PICO question

| **Population** | **Intervention** | **Phenomenon** |
| --- | --- | --- |
| **(Health Personnel[Mesh]** OR Health Personnel[tiab] OR Healthcare Personnel[tiab] OR Health Worker*[tiab] OR Nurse*[tiab] OR Doctor*[tiab] OR Physician*[tiab] OR Paramedic*[tiab] OR Caregiver*[tiab] OR Care Giver*[tiab] OR Medic*[tiab) | **(Education, Distance[Mesh]** OR Distance Education[tiab] OR Online Education[tiab] OR ELearning[tiab] OR Learning[tiab] OR Workshop*[tiab] OR Training[tiab]) | **(Disasters[Mesh]** OR Disaster*[tiab] OR Catastroph*[tiab] OR Mass Casualt*[tiab] OR Terroris*[tiab] OR Bioterroris*[tiab] OR **Epidemics[Mesh**] OR Epidemic*[tiab] OR Pandemic*[tiab] OR Natural Hazard*[tiab] OR Humanitarian Emergen*[tiab] OR Avalanche*[tiab] OR Storm*[tiab] OR Cyclon*[tiab] OR Drought*[tiab] OR Hurricane*[tiab] OR Typhoon*[tiab] OR Earthquake*[tiab] OR Volcanic*[tiab] OR **Volcanic Eruptions**[Mesh] OR Volcanic*[tiab] OR Volcano*[tiab] OR Tsunami*[tiab] OR Flood*[tiab] OR Wildfire*[tiab] OR Wild-Fire*[tiab] OR Wildfire*[tiab] OR Bushfire*[tiab] OR Landslide*[tiab] OR Industrial Accident*[tiab] OR Chemical Hazard*[tiab] OR Chemical Emergenc*[tiab] OR Nuclear Hazard*[tiab] OR Nuclear Emegenc*[tiab] OR Radiological Hazard*[tiab] OR Radiological Emergenc*[tiab] OR Biological Hazard*[tiab] OR Biological Emergenc*[tiab] OR Biohazard*[tiab] OR Warfare*[tiab] OR Armed Conflict*[tiab] OR War[tiab]) |

Search strategy

Pubmed search

| **Search** | **Actions** | **Details** | **Query** | **Results** | **Time** |
| --- | --- | --- | --- | --- | --- |
| #4 |  |  | **#1 AND #2 AND #3** | [14,186](https://pubmed.ncbi.nlm.nih.gov/?term=%231+AND+%232+AND+%233&sort=) | 10:41:57 |
| #3 |  |  | **(Disasters[Mesh] OR Disaster*[tiab] OR Catastroph*[tiab] OR Mass Casualt*[tiab] OR Terroris*[tiab] OR Bioterroris*[tiab] OR Epidemics[Mesh] OR Epidemic*[tiab] OR Pandemic*[tiab] OR Natural Hazard*[tiab] OR Humanitarian Emergen*[tiab] OR Avalanche*[tiab] OR Storm*[tiab] OR Cyclon*[tiab] OR Drought*[tiab] OR Hurricane*[tiab] OR Typhoon*[tiab] OR Earthquake*[tiab] OR Volcanic*[tiab] OR Volcanic Eruptions[Mesh] OR Volcanic*[tiab] OR Volcano*[tiab] OR Tsunami*[tiab] OR Flood*[tiab] OR Wildfire*[tiab] OR Wild-Fire*[tiab] OR Wildfire*[tiab] OR Bushfire*[tiab] OR Landslide*[tiab] OR Industrial Accident*[tiab] OR Chemical Hazard*[tiab] OR Chemical Emergenc*[tiab] OR Nuclear Hazard*[tiab] OR Nuclear Emegenc*[tiab] OR Radiological Hazard*[tiab] OR Radiological Emergenc*[tiab] OR Biological Hazard*[tiab] OR Biological Emergenc*[tiab] OR Biohazard*[tiab] OR Warfare*[tiab] OR Armed Conflict*[tiab] OR War[tiab])** | 603,360 | 10:41:20 |
| #2 |  |  | Search: **(Education, Distance[Mesh] OR Distance Education[tiab] OR Online Education[tiab] OR ELearning[tiab] OR Learning[tiab] OR Workshop*[tiab] OR Training[tiab])** | [936,830](https://pubmed.ncbi.nlm.nih.gov/?term=%28Education%2C+Distance%5BMesh%5D+OR+Distance+Education%5Btiab%5D+OR+Online+Education%5Btiab%5D+OR+ELearning%5Btiab%5D+OR+Learning%5Btiab%5D+OR+Workshop%2A%5Btiab%5D+OR+Training%5Btiab%5D%29&sort=) | 10:39:18 |
| #1 |  |  | \| Search: **(Health Personnel[Mesh] OR Health Personnel[tiab] OR Healthcare Personnel[tiab] OR Health Worker*[tiab] OR Nurse*[tiab] OR Doctor*[tiab] OR Physician*[tiab] OR Paramedic*[tiab] OR Caregiver*[tiab] OR Care Giver*[tiab] OR Medic*[tiab)** \| \| --- \| | \| [3,385,086](https://pubmed.ncbi.nlm.nih.gov/?term=%28Health+Personnel%5BMesh%5D+OR+Health+Personnel%5Btiab%5D+OR+Healthcare+Personnel%5Btiab%5D+OR+Health+Worker%2A%5Btiab%5D+OR+Nurse%2A%5Btiab%5D+OR+Doctor%2A%5Btiab%5D+OR+Physician%2A%5Btiab%5D+OR+Paramedic%2A%5Btiab%5D+OR+Caregiver%2A%5Btiab%5D+OR+Care+Giver%2A%5Btiab%5D+OR+Medic%2A%5Btiab%29&sort=) \| 10:38:55 \| \| --- \| --- \| |  |

The filter for Low and Middle-income countries was then applied. The final search strategy is presented below:

**Full Strategy:**

**(Disasters[Mesh]** OR Disaster*[tiab] OR Catastroph*[tiab] OR Mass Casualt*[tiab] OR Terroris*[tiab] OR Bioterroris*[tiab] OR **Epidemics[Mesh**] OR Epidemic*[tiab] OR Pandemic*[tiab] OR Natural Hazard*[tiab] OR Humanitarian Emergen*[tiab] OR Avalanche*[tiab] OR Storm*[tiab] OR Cyclon*[tiab] OR Drought*[tiab] OR Hurricane*[tiab] OR Typhoon*[tiab] OR Earthquake*[tiab] OR Volcanic*[tiab] OR **Volcanic Eruptions**[Mesh] OR Volcanic*[tiab] OR Volcano*[tiab] OR Tsunami*[tiab] OR Flood*[tiab] OR Wildfire*[tiab] OR Wild-Fire*[tiab] OR Wildfire*[tiab] OR Bushfire*[tiab] OR Landslide*[tiab] OR Industrial Accident*[tiab] OR Chemical Hazard*[tiab] OR Chemical Emergenc*[tiab] OR Nuclear Hazard*[tiab] OR Nuclear Emegenc*[tiab] OR Radiological Hazard*[tiab] OR Radiological Emergenc*[tiab] OR Biological Hazard*[tiab] OR Biological Emergenc*[tiab] OR Biohazard*[tiab] OR Warfare*[tiab] OR Armed Conflict*[tiab] OR War[tiab]) **AND** **(Health Personnel[Mesh]** OR Health Personnel[tiab] OR Healthcare Personnel[tiab] OR Health Worker*[tiab] OR Nurse*[tiab] OR Doctor*[tiab] OR Physician*[tiab] OR Paramedic*[tiab] OR Caregiver*[tiab] OR Care Giver*[tiab] OR Medic*[tiab) **AND** **(Education, Distance[Mesh]** OR Distance Education[tiab] OR Online Education[tiab] OR ELearning[tiab] OR Learning[tiab] OR Workshop*[tiab] OR Training[tiab]) **AND** (LMIC[tiab] OR "Low and Middle"[tiab] OR Subsaharian[tiab] OR Sub Saharian[tiab] OR Southeast Asia*[tiab] OR Middle East*[tiab] OR Central America*[tiab] OR Africa[Mesh] OR Afghanistan[Mesh] OR Afghan*[tiab] OR Benin[Mesh]OR Benin*[tiab] OR Burkina Faso[Mesh] OR Burkin*[tiab] OR Burundi[Mesh] OR Burundi*[tiab] OR Central African Republic[Mesh] OR Central African[tiab] OR Chad[Mesh] OR Chad[tiab] OR Albania[Mesh] OR Albania*[tiab] OR Angola[Mesh] OR Angola[tiab] OR Algeria[Mesh] OR Algeria*[tiab] OR Armenia[Mesh] OR Armenia*[tiab] OR Azerbaijan[Mesh] OR Azerbaijan*[tiab] OR Bangladesh[Mesh] OR Bangladesh*[tiab] OR Republic of Belarus[Mesh] OR Belarus*[tiab] OR Belize[Mesh] OR Beliz*[tiab] OR Bhutan[Mesh] OR Bhutan*[tiab] OR Bolivia[Mesh] OR Bolivia*[tiab] OR “Bosnia and Herzegovina”[Mesh] OR Bosni*[tiab] OR Botswana[Mesh] OR Botswan*[tiab] OR Brazil[Mesh] OR Brazil*[tiab] OR Bulgaria[Mesh] OR Bulgaria*[tiab] OR Cabo Verde[Mesh] OR Cabo Verde*[tiab] OR Cambodia[Mesh] OR Cambodia*[tiab] OR Cameroon[Mesh] OR Cameroon*[tiab] OR China[Mesh] OR China[tiab] OR Chinese[tiab] OR Colombia[Mesh] OR Colombia*[tiab] OR Comoros[Mesh] OR Comoro*[tiab] OR Democratic Republic of the Congo[Mesh] OR Congo*[tiab] OR Costa Rica[Mesh] OR Costa Rica[tiab] OR Costarica*[tiab] OR Cote d'Ivoire[Mesh] OR “Côte d'Ivoire”[tiab] OR Cuba[Mesh] OR Cuba*[tiab] OR Djibouti[Mesh] OR Djibout*[tiab] OR Dominican Republic[Mesh] OR Dominic*[tiab] OR Ecuador[Mesh] OR Ecuador*[tiab] OR Egypt[Mesh] OR Egypt*[tiab] OR El Salvador[Mesh] OR Salvador*[tiab] OR Eritrea[Mesh]OR Eritrea*[tiab] OR Ethiopia[Mesh] OR Ethiopi*[tiab] OR Fiji[Mesh] OR Fiji*[tiab] OR Gabon[Mesh] OR Gabon*[tiab] OR Gambia[Mesh] OR Gambia*[tiab] OR “Georgia (Republic)”[Mesh] OR Georgia*[tiab] OR Ghana[Mesh] OR Ghana*[tiab] OR Guatemala[Mesh] OR Guatemal*[tiab] OR Guinea[Mesh] OR Guinea-Bissau[Mesh] OR Guinea*[tiab] OR Guyana[Mesh] OR Guyan*[tiab] OR Gabon OR Haiti[Mesh] OR Haiti*[tiab] OR Honduras[Mesh] OR Hondur*[tiab] OR India[Mesh] OR India[tiab] OR Indonesia[Mesh] OR Indones*[tiab] OR Iran[Mesh] OR Iran*[tiab] OR Iraq[Mesh] OR Iraq[tiab] OR Jamaica[Mesh] OR Jamai*[tiab] OR Jordan[Mesh] OR Jordan*[tiab] OR Kazakhstan[Mesh] OR Kazakhstan*[tiab] OR Kenya[Mesh] OR Kenya*[tiab] OR Micronesia[Mesh] OR Micronesia*[tiab] OR Kiribati*[tiab] OR Kosovo[Mesh] OR Kosov*[tiab] OR Kyrgyzstan[Mesh] OR Kyrgyzstan*[tiab] OR “Democratic People's Republic of Korea”[Mesh] OR North Korea*[tiab] OR Laos[Mesh] OR Laos*[tiab] OR Lebanon[Mesh] OR Leban*[tiab] OR Lesotho[Mesh] OR Lesoth*[tiab] OR Liberia[Mesh] OR Liberia*[tiab] OR Libya[Mesh] OR Libya*[tiab] OR “Macedonia (Republic)”[Mesh] OR Macedonia*[tiab] OR Madagascar[Mesh] OR Madagascar*[tiab] OR Malawi[Mesh] OR Malawi*[tiab] OR Mali[Mesh] OR Mali[tiab] OR Mauritania[Mesh] OR Mauritan*[tiab] OR Mauritius[Mesh] OR Mauriti*[tiab] OR Mexico[Mesh] OR Mexic*[tiab] OR Moldova[Mesh] OR Moldov*[tiab] OR Mongolia[Mesh] OR Mongolia*[tiab] OR Montenegro[Mesh] OR Montenegr*[tiab] OR Morocco[Mesh] OR Morocc*[tiab] OR Myanmar[Mesh] OR Myanmar*[tiab] OR Mozambique[Mesh] OR Mozambiq*[tiab] OR Namibia[Mesh] OR Namibia*[tiab] OR Nepal[Mesh] OR Nepal*[tiab] OR Nicaragua[Mesh] OR Nicaragu*[tiab] OR Niger[Mesh] OR Niger*[tiab] OR Nigeria[Mesh] OR Nigeri*[tiab] OR Pakistan[Mesh] OR Pakistan*[tiab] OR Palau[Mesh] OR Palau*[tiab] OR Panama[Mesh] OR Panam*[tiab] OR Papua New Guinea[Mesh] OR Papua*[tiab] OR Paraguay[Mesh] OR Paraguay*[tiab] OR Peru[Mesh] OR Peru*[tiab] OR Philippines[Mesh] OR Philippine*[tiab] OR Rwanda[Mesh] OR Rwand*[tiab] OR Samoa[Mesh] OR Samoa*[tiab] OR Senegal[Mesh] OR Senegal*[tiab] OR Serbia[Mesh] OR Serbia*[tiab] OR Sierra Leone[Mesh] OR Sierra Leon*[tiab] OR Somalia[Mesh] OR Somalia*[tiab] OR South Africa[Mesh] OR South Africa[tiab] OR Southafrica*[tiab] OR Sri Lanka[Mesh] OR Sri Lanka*[tiab] OR Swaziland[Mesh] OR Swaziland*[tiab] OR Sudan[Mesh] OR Sudan*[tiab] OR Syria[Mesh] OR Syria*[tiab] OR Tajikistan[Mesh] OR Tajikistan*[tiab] OR Tanzania[Mesh] OR Tanzania*[tiab] OR Thailand[Mesh] OR Thailand*[tiab] OR Timor-Leste[Mesh] OR Timor*[tiab] OR Tonga[Mesh] OR Tonga*[tiab] OR Togo[Mesh] OR Togo*[tiab] OR Tunisia[Mesh] OR Tunisia*[tiab] OR Turkey[Mesh] OR Turk*[tiab] OR Turkmenistan[Mesh] OR Ukraine[Mesh] OR Ukrain*[tiab] OR Uganda[Mesh] OR Ugand*[tiab] OR Uzbekistan[Mesh] OR Uzbekistan*[tiab] OR Venezuela[Mesh] OR Venezuel*[tiab] OR Vietnam[Mesh] OR Vietnam*[tiab] OR Yemen[Mesh] OR Yemen*[tiab] OR Zambia[Mesh] OR Zambia*[tiab] OR Zimbabwe[Mesh] OR Zimbabwe*[tiab])

Embase search

**(Disasters[Mesh]** OR Disaster*[tiab] OR Catastroph*[tiab] OR Mass Casualt*[tiab] OR Terroris*[tiab] OR Bioterroris*[tiab] OR **Epidemics[Mesh**] OR Epidemic*[tiab] OR Pandemic*[tiab] OR Natural Hazard*[tiab] OR Humanitarian Emergen*[tiab] OR Avalanche*[tiab] OR Storm*[tiab] OR Cyclon*[tiab] OR Drought*[tiab] OR Hurricane*[tiab] OR Typhoon*[tiab] OR Earthquake*[tiab] OR **Volcanic Eruptions**[Mesh] OR Volcanic*[tiab] OR Volcano*[tiab] OR Tsunami*[tiab] OR Flood*[tiab] OR Wildfire*[tiab] OR Wild-Fire*[tiab] OR Bushfire*[tiab] OR Landslide*[tiab] OR Industrial Accident*[tiab] OR Chemical Hazard*[tiab] OR Chemical Emergenc*[tiab] OR Nuclear Hazard*[tiab] OR Nuclear Emergenc*[tiab] OR Radiological Hazard*[tiab] OR Radiological Emergenc*[tiab] OR Biological Hazard*[tiab] OR Biological Emergenc*[tiab] OR Biohazard*[tiab] OR Warfare*[tiab] OR Armed Conflict*[tiab] OR War[tiab]) **AND** **(Health Personnel[Mesh]** OR Health Personnel[tiab] OR Healthcare Personnel[tiab] OR Health Worker*[tiab] OR Nurse*[tiab] OR Doctor*[tiab] OR Physician*[tiab] OR Paramedic*[tiab] OR Caregiver*[tiab] OR Care Giver*[tiab] OR Medic*[tiab) **AND** **(Education, Distance[Mesh]** OR Distance Education[tiab] OR Online Education[tiab] OR ELearning[tiab] OR Learning[tiab] OR Workshop*[tiab] OR Training[tiab]) AND ( )

Embase Classic+Embase <2003 to 2023 February 03>

1 exp disaster/ 34462

2 Disaster*.ti,ab. 36012

3 Catastroph*.ti,ab. 42907

4 (Mass adj1 Casualt*).ti,ab. 3249

5 Terroris*.ti,ab. 8344

6 Bioterroris*.ti,ab. 4279

7 exp epidemic/ 143913

8 Epidemic*.ti,ab. 161539

9 Pandemic*.ti,ab. 208701

10 (Natural adj1 Hazard*).ti,ab. 805

11 (Humanitarian adj1 Emergen*).ti,ab. 387

12 Avalanche*.ti,ab. 3211

13 Storm*.ti,ab. 29420

14 Cyclon*.ti,ab. 3947

15 Drought*.ti,ab. 26557

16 Hurricane*.ti,ab. 4734

17 Typhoon*.ti,ab. 963

18 Earthquake*.ti,ab. 10619

19 Volcanic*.ti,ab. 5178

20 exp volcano/ 4481

21 Volcano*.ti,ab. 3793

22 Tsunami*.ti,ab. 3274

23 Flood*.ti,ab. 24313

24 Wildfire*.ti,ab. 3279

25 Wild-Fire*.ti,ab. 103

26 Bushfire*.ti,ab. 507

27 Landslide*.ti,ab. 1021

28 (Industrial adj1 Accident*).ti,ab. 1735

29 (Chemical adj1 Hazard*).ti,ab. 1766

30 (Chemical adj1 Emergenc*).ti,ab. 117

31 (Nuclear adj1 Hazard*).ti,ab. 45

32 (Nuclear adj1 Emergenc*).ti,ab. 353

33 (Radiological adj1 Hazard*).ti,ab. 538

34 (Radiological adj1 Emergenc*).ti,ab. 385

35 (Biological adj1 Hazard*).ti,ab. 709

36 (Biological adj1 Emergenc*).ti,ab. 46

37 Biohazard*.ti,ab. 1173

38 Warfare*.ti,ab. 6504

39 (Armed adj1 Conflict*).ti,ab. 1769

40 War.ti,ab. 49432

41 or/1-40 671661

42 exp health care personnel/ 1983234

43 (Health* adj3 Personnel).ti,ab. 15912

44 (Health* adj3 Worker*).ti,ab. 85974

45 Nurse*.ti,ab. 391356

46 Doctor*.ti,ab. 220614

47 Physician*.ti,ab. 660892

48 Medics.ti,ab. 1620

49 Paramedic*.ti,ab. 14540

50 Caregiver*.ti,ab. 119233

51 (Care adj1 Giver*).ti,ab. 5438

52 or/42-51 2674948

53 exp distance learning/ 5901

54 (Distance adj1 Education).ti,ab. 1206

55 (Online adj1 Education).ti,ab. 1980

56 ELearning.ti,ab. 806

57 Learning.ti,ab. 554760

58 Workshop*.ti,ab. 65971

59 Training.ti,ab. 711671

60 or/53-59 1213645

61 (LMIC or "Low and Middle" or Subsaharian or "Sub Saharian" or "Southeast Asia*" or "Middle East*" or "Central America*").tw. or exp Africa/ or exp Afghanistan/ or Afghan*.tw. or exp Benin/ or exp "Burkina Faso"/ or Burkin*.tw. or exp Burundi/ or Burundi*.tw. or exp "Central African Republic"/ or "Central African".tw. or exp Chad/ or Chad.tw. or exp Albania/ or Albania*.tw. or exp Angola/ or Angola.tw. or exp Algeria/ or Algeria*.tw. or exp Armenia/ or Armenia*.tw. or exp Azerbaijan/ or Azerbaijan*.tw. or exp Bangladesh/ or Bangladesh*.tw. or exp "Republic of Belarus"/ or Belarus*.tw. or exp Belize/ or Beliz*.tw. or exp Bhutan/ or Bhutan*.tw. or exp Bolivia/ or Bolivia*.tw. or exp "Bosnia and Herzegovina"/ or Bosni*.tw. or exp Botswana/ or Botswan*.tw. or exp Brazil/ or Brazil*.tw. or exp Bulgaria/ or Bulgaria*.tw. or exp "Cabo Verde"/ or "Cabo Verde*".tw. or exp Cambodia/ or Cambodia*.tw. or exp Cameroon/ or Cameroon*.tw. or exp China/ or China.tw. or Chinese.tw. or exp Colombia/ or Colombia*.tw. or exp Comoros/ or Comoro*.tw. or exp "Democratic Republic of the Congo"/ or Congo*.tw. or exp "Costa Rica"/ or "Costa Rica".tw. or Costarica*.tw. or exp "Cote d'Ivoire"/ or "Côte d'Ivoire".tw. or exp Cuba/ or Cuba*.tw. or exp Djibouti/ or Djibout*.tw. or exp "Dominican Republic"/ or Dominic*.tw. or exp Ecuador/ or Ecuador*.tw. or exp Egypt/ or Egypt*.tw. or exp "El Salvador"/ or Salvador*.tw. or exp Eritrea/ or exp Ethiopia/ or Ethiopi*.tw. or exp Fiji/ or Fiji*.tw. or exp Gabon/ or Gabon*.tw. or exp Gambia/ or Gambia*.tw. or exp "Georgia (Republic)"/ or Georgia*.tw. or exp Ghana/ or Ghana*.tw. or exp Guatemala/ or Guatemal*.tw. or exp Guinea/ or exp Guinea-Bissau/ or Guinea*.tw. or exp Guyana/ or Guyan*.tw. or Gabon.mp. or exp Haiti/ or Haiti*.tw. or exp Honduras/ or Hondur*.tw. or exp India/ or India.tw. or exp Indonesia/ or Indones*.tw. or exp Iran/ or Iran*.tw. or exp Iraq/ or Iraq.tw. or exp Jamaica/ or Jamai*.tw. or exp Jordan/ or Jordan*.tw. or exp Kazakhstan/ or Kazakhstan*.tw. or exp Kenya/ or Kenya*.tw. or exp Micronesia/ or Micronesia*.tw. or Kiribati*.tw. or exp Kosovo/ or Kosov*.tw. or exp Kyrgyzstan/ or Kyrgyzstan*.tw. or exp "Democratic People's Republic of Korea"/ or "North Korea*".tw. or exp Laos/ or Laos*.tw. or exp Lebanon/ or Leban*.tw. or exp Lesotho/ or Lesoth*.tw. or exp Liberia/ or Liberia*.tw. or exp Libya/ or Libya*.tw. or exp "Macedonia (Republic)"/ or Macedonia*.tw. or exp Madagascar/ or Madagascar*.tw. or exp Malawi/ or Malawi*.tw. or exp Mali/ or Mali.tw. or exp Mauritania/ or Mauritan*.tw. or exp Mauritius/ or Mauriti*.tw. or exp Mexico/ or Mexic*.tw. or exp Moldova/ or Moldov*.tw. or exp Mongolia/ or Mongolia*.tw. or exp Montenegro/ or Montenegr*.tw. or exp Morocco/ or Morocc*.tw. or exp Myanmar/ or Myanmar*.tw. or exp Mozambique/ or Mozambiq*.tw. or exp Namibia/ or Namibia*.tw. or exp Nepal/ or Nepal*.tw. or exp Nicaragua/ or Nicaragu*.tw. or exp Niger/ or Niger*.tw. or exp Nigeria/ or Nigeri*.tw. or exp Pakistan/ or Pakistan*.tw. or exp Palau/ or Palau*.tw. or exp Panama/ or Panam*.tw. or exp "Papua New Guinea"/ or Papua*.tw. or exp Paraguay/ or Paraguay*.tw. or exp Peru/ or Peru*.tw. or exp Philippines/ or Philippine*.tw. or exp Rwanda/ or Rwand*.tw. or exp Samoa/ or Samoa*.tw. or exp Senegal/ or Senegal*.tw. or exp Serbia/ or Serbia*.tw. or exp "Sierra Leone"/ or "Sierra Leon*".tw. or exp Somalia/ or Somalia*.tw. or exp "South Africa"/ or "South Africa".tw. or Southafrica*.tw. or exp "Sri Lanka"/ or "Sri Lanka*".tw. or exp Swaziland/ or Swaziland*.tw. or exp Sudan/ or Sudan*.tw. or exp Syria/ or Syria*.tw. or exp Tajikistan/ or Tajikistan*.tw. or exp Tanzania/ or Tanzania*.tw. or exp Thailand/ or Thailand*.tw. or exp Timor-Leste/ or Timor*.tw. or exp Tonga/ or Tonga*.tw. or exp Togo/ or Togo*.tw. or exp Tunisia/ or Tunisia*.tw. or exp Turkey/ or Turk*.tw. or exp Turkmenistan/ or exp Ukraine/ or Ukrain*.tw. or exp Uganda/ or Ugand*.tw. or exp Uzbekistan/ or Uzbekistan*.tw. or exp Venezuela/ or Venezuel*.tw. or exp Vietnam/ or Vietnam*.tw. or exp Yemen/ or Yemen*.tw. or exp Zambia/ or Zambia*.tw. or exp Zimbabwe/ or Zimbabwe*.tw. [mp=title, abstract, heading word, drug trade name, original title, device manufacturer, drug manufacturer, device trade name, keyword heading word, floating subheading word, candidate term word] 2590631

62 41 and 52 and 60 and 61 3398

Table 3: Type of study design

| *Study design* | N |
| --- | --- |
| Descriptive | 173 |
| Before-after | 87 |
| RCT | 18 |
| Cross-sectional | 14 |
| Observational | 12 |
| Review | 7 |
| Qualitative | 5 |
| Opinion | 3 |
| **Total** | **319** |

Table 4: Learner distribution across studies

| *Learner* | N |
| --- | --- |
| Health workforce | 268 |
| Military | 13 |
| Health workforce and community | 12 |
| Citizens and affected population | 12 |
| Experts and volunteers | 9 |
| National Institutions | 4 |
| Academia | 1 |
| **Total** | **319** |
