# Supplementary material for: Learning interventions and training methods in health emergencies: A scoping review
Source: PLoS One. 2024 Jul 16;19(7):e0290208. doi: 10.1371/journal.pone.0290208 (PMC11251632; doi:10.1371/journal.pone.0290208)
Supplement: Attachment — Submitted filename: plos review.docx [file pone.0290208.s002.docx]

**General:**

*Overall, an important research question, but the paper at this time lacks finesse in terms of critical analysis and conducting a scoping review properly. If truly global, in reference to what the WHO wished to discover, I feel you hindered yourselves by using too many search terms as you did, in terms of naming all the countries in regions you focused on. More appropriate and probably less search terms would have found more papers, especially if you used more than 2 databases.*

*As noted below, just 2 databases for a scoping review weakens your findings. Your findings are not analysed in depth and this is highlighted in the discussion in terms of no clear type of analysis of the papers (e.g thematic, narrative etc), critical analysis compared to other literature.*

*With more work, this review would be incredibly valuable, but it needs quite a bit work at this stage. This is especially the case, as, for a scoping review it has a low number of database sources. Some general comments below, followed by some specific ones:*

- Do not discuss the big elephant in the room – lack of accreditation (i.e university level standard of training or royal college standard). WHO standard is only a guide and does not meet the QA of training that universities provide – surely, emergency training must be meet this standard? This can be addressed in the introduction and discussion. Would standards, e.g a term such as ‘accredited’ affected your search, and possibly made for a different, but more accurate paper?
- A weakness is that only 2 databases were used, for a scoping review more databases should have been utilized. Additionally, grey literature should have been considered, considering the WHO involvement. 2 databases would be fine if it were a systematic review.
- In your methodology, you state you are ‘analyzing’ and ‘developing an understanding’, for this review to have merit, your analysis should be either narrative but ideally thematic in nature and this is not clear. Line 280 says ‘groupings’ which is not very scientific, your analysis structure needs to be clearer.
- Major topic areas – you do not go into enough detail. You need to describe the papers well, with data, geography and numbers. It comes across as too broad and not specific in terms of analysis.
- Table of papers should be included in the main body of the paper, not as a supplement
- Change your referencing style if possible
- Your discussion, though touching on your findings, does not explore the literature in terms of the ‘major topic areas’ you found – what else is discussed in the literature around this? E.g MOOCs are considered poor educational tools, how does this compare with your findings? AI use, what is currently being pubished and found,, compared to your findings? This highlights a lack of critical analysis in the discussion and this needs to be addressed.

**Specific:**

- Line 24: ‘the latest evolving knowledge’ – knowledge of what? Needs to be rewritten.
- Line 43: Abstract results should be more detailed in terms of types of papers used and in brackets original findings to what what was analysed
- Line 60-72: More definition around what emergencies are and what is referred to as system strengthening – the reader should be able to understand why, though in general why certain systems need strengthening – whether this be due to lack of infrastructure or more importantly no UHC, with private healthcare hindering responses
- Line 89 – the ‘ongoing’ covid 19 pandemic, do not assume it is over or refer to it in the past tense
- Line 233: Location paragraph needs work: 3 countries only? Yet, your table has papers from Lebanon and other countries in sub Saharan Africa. This needs to be corrected and be more detailed. (Again due to the weakness of databases and search, most papers were from 3 geographies, China, India and South Africa. You mention this is due to covid yet, your search time frame was from 2003, this does not make sense and needs a re-evaluation in terms of papers and search.)
- Figure 3: percentages do not add up to 100%, instead 99.3 (strongly advised against the use of pie charts, bar charts are more appropriate). Plus legends of figures need to be more detailed
