# Supplementary material for: Learning interventions and training methods in health emergencies: A scoping review
Source: PLoS One. 2024 Jul 16;19(7):e0290208. doi: 10.1371/journal.pone.0290208 (PMC11251632; doi:10.1371/journal.pone.0290208)
Supplement: Attachment — Submitted filename: Answer to the reviewer.docx [file pone.0290208.s003.docx]

Dear reviewer:

We appreciate your comments and very valuable inputs to our work. We hereby submit our responses below:

**General Comments:**

1) Do not discuss the big elephant in the room – lack of accreditation (i.e university level standard of training or royal college standard). WHO standard is only a guide and does not meet the QA of training that universities provide – surely, emergency training must be meet this standard? This can be addressed in the introduction and discussion. Would standards, e.g a term such as ‘accredited’ affected your search, and possibly made for a different, but more accurate paper?

Answer: Our scoping review focuses mainly on just in time learning and professional accreditation is excluded. We further clarified inclusion and exclusion criteria in line 152.

2) A weakness is that only 2 databases were used, for a scoping review more databases should have been utilized. Additionally, grey literature should have been considered, considering the WHO involvement.

Answer: This scoping review included only peer reviewed literature. WHO has done a grey literature review and will be published in a separate article. We expanded the search to HINARI, Web of Science, WorldCat and CABI and updated the results accordingly.

3) In your methodology, you state you are ‘analyzing’ and ‘developing an understanding’, for this review to have merit, your analysis should be either narrative but ideally thematic in nature and this is not clear. Line 280 says ‘groupings’ which is not very scientific, your analysis structure needs to be clearer.

Answer: To avoid confusion, we have removed mention of analyzing and grouping data and reverted to using Arskey and O’Malley’ exact language of collating/summarizing. We present the results as an evidence map. We then summarized the studies by major topic areas and briefly described key aspects of the studies under these headings. A recent similar scoping review using Arskey and O’Malley’s methodology publish by PLOS ONE can be found here: <https://pubmed.ncbi.nlm.nih.gov/36827258/>. Of note McGill’s scoping review included far fewer studies (86) compared with our review (319).

4) Major topic areas – you do not go into enough detail. You need to describe the papers well, with data, geography and numbers. It comes across as too broad and not specific in terms of analysis.

Answer: This scoping review included a huge number of studies (319). A lot of detail at individual study level can be found in the included study table. The visual displays provide additional collated information. Within the limited scope of collating/summarizing of Arskey and O’Malley’s scoping review methodology, there is not a methodological requirement to describe the studies in detail and given the huge number of studies it would not have been feasible to do so. As stated in line 126 the objective of this scoping review is to map the evidence so that subsequently review questions can be identified and subsequent systematic reviews can be commissioned by WHO.

5) Table of papers should be included in the main body of the paper, not as a supplement.

Answer: It has been moved to line 232. The table reporting 319 studies is however enormous. We welcome any feedback from the managing Editor as to whether this table is too big to include in the body of the main manuscript.

6) Change your referencing style if possible.

Answer: Referencing style is Vancouver, suggested by the journal. If there is another referencing style we should use, please let us know and we will change it.

7) Your discussion, though touching on your findings, does not explore the literature in terms of the ‘major topic areas’ you found – what else is discussed in the literature around this? E.g MOOCs are considered poor educational tools, how does this compare with your findings? AI use, what is currently being pubished and found,, compared to your findings? This highlights a lack of critical analysis in the discussion and this needs to be addressed.

Answer: As stated in line 126, the objective of this scoping review was to map the existing evidence on the topic. We decided to undertake a scoping review (and not a systematic review) in order to map 319 included studies. Scoping reviews are generally not designed to critically evaluate the content or results of included studies or situate what was found in the context of broader literature.

**Specific Comments:**

1) Line 24: ‘the latest evolving knowledge’ – knowledge of what? Needs to be rewritten.

Answer: We rewrote the paragraph

2) Line 43: Abstract results should be more detailed in terms of types of papers used and in brackets original findings to what was analysed.

Answer: The PLOS ONE abstract is limited to 300 words. According to the PLOS ONE author guidance, ‘The Abstract should be succinct; it must not exceed 300 words. Authors should mention the techniques used without going into methodological detail and should summarize the most important results without going into detail’. Our abstract is currently 325 words and we therefore need to remove words and further cut back on detail rather than add.

3) Line 60-72: More definition around what emergencies are and what is referred to as system strengthening – the reader should be able to understand why, though in general why certain systems need strengthening – whether this be due to lack of infrastructure or more importantly no UHC, with private healthcare hindering responses

Answer: We rewrote the introduction.

4) Line 89 – the ‘ongoing’ covid 19 pandemic, do not assume it is over or refer to it in the past tense Changed the verbal term to present

Answer: We rewrote the introduction.

5) Line 233: Location paragraph needs work: 3 countries only? Yet, your table has papers from Lebanon and other countries in sub Saharan Africa. This needs to be corrected and be more detailed. (Again due to the weakness of databases and search, most papers were from 3 geographies, China, India and South Africa. You mention this is due to covid yet, your search time frame was from 2003, this does not make sense and needs a re-evaluation in terms of papers and search.)

Answer: We rewrote the paragraph in line 252 and added a figure

- Figure 3: percentages do not add up to 100%, instead 99.3 (strongly advised against the use of pie charts, bar charts are more appropriate). Plus legends of figures need to be more detailed

Answer: We corrected the figure, now it is figure 4.
