# Supplementary material for: Learning interventions and training methods in health emergencies: A scoping review
Source: PLoS One. 2024 Jul 16;19(7):e0290208. doi: 10.1371/journal.pone.0290208 (PMC11251632; doi:10.1371/journal.pone.0290208)
Supplement: Attachment — Submitted filename: Final Response to Reviewers .docx [file pone.0290208.s004.docx]

Dear reviewers:

We appreciate your comments and very valuable inputs to our work. We hereby submit our responses below.

**Major comment to the authors from the Reviewer 1:**

Thank you to the three peer reviewers for reviewing the revised manuscript. We note that Reviewer 2 and Reviewer 3 are satisfied with the format, methods and content of the scoping review and the comments received have been actioned on from our side. These two reviewers conclude this scoping review reported in the manuscript follows a similar purpose and format to scoping reviews that we have previously published in this journal(1), and others have published in this journal using the same methodology(2).

REVIEWER 1 COMMENT:

Using a scoping review as 'limited in purpose' as a limitation, excuses the lack of any in depth analysis. All types of reviews have limitations, however some of the most successful and impactful scoping reviews have an in-depth analysis. This a real shame. The topic and paper overall is an interesting read, but, lack of proper analysis. This is reflected in the discussion.

RESPONSE: We acknowledge that reviewer 1 is requesting more clarifications to the manuscript including a critical analysis of the literature.  We have addressed our concerns about critical analysis in the general comments and we have updated our discussion in line 460.

We have included three extracts here from recent seminal methodological works on the purpose of a scoping review as a sample no mention of a PRISMA for scoping reviews(3), beyond charting and describing, additional analyses are not required. Critical appraisal of the included sources has also been optional in other works(4, 5). We therefore suggest this manuscript to be processed as is, without the additional more-in-depth critical analysis of the literature and hope this is an agreeable way forward, in light of the referenced work and in line with the Reviewers 2 and 3 who are in favor of our manuscript.

**Detailed comments to the Authors**

COMMENT Figure 3 is unfortunately not of any use – pie charts in general are not ideal for this amount of data.

RESPONSE We have deleted figure number 3, all necessary information can be found in the summary of studies table.

COMMENT: Only two databases are mentioned in the methods section of the abstract (line 40-41). They authors should consider updating this.

RESPONSE This has been updated in line 42 by adding the full database search.

COMMENT The authors should consider including a comprehensive list of abbreviation

RESPONSE Abbreviations has been added following PLOS one guidelines.

COMMENT The authors should consider including the rationale for excluding grey literature from their review in the body of the text.

RESPONSE: This issue has been addressed in line 195 by adding the justification for the exclusion of grey literature from this paper.

References:

1.	Silveira Bianchim M., Crane E., Jones A., Neukirchinger B., Roberts G., McLaughlin L., Noyes J. The implementation, use and impact of patient reported outcome measures in value-based healthcare programmes: A scoping review. PLoS One. 2023;18(12):e0290976.

2.	McGill E., Halliday E., Egan M., Popay J. Knowledge exchange in crisis settings: A scoping review. PLoS One. 2023;18(2):e0282080.

3.	Tricco A. C., Lillie E., Zarin W., O'Brien K. K., Colquhoun H., Levac D., Moher D., Peters M. D. J., Horsley T., Weeks L., Hempel S., Akl E. A., Chang C., McGowan J., Stewart L., Hartling L., Aldcroft A., Wilson M. G., Garritty C., Lewin S., et al. PRISMA Extension for Scoping Reviews (PRISMA-ScR): Checklist and Explanation. Ann Intern Med. 2018;169(7):467-73.

4.	Peters M. D. J., Marnie C., Colquhoun H., Garritty C. M., Hempel S., Horsley T., Langlois E. V., Lillie E., O'Brien K. K., Tuncalp Ӧ, Wilson M. G., Zarin W., Tricco A. C. Scoping reviews: reinforcing and advancing the methodology and application. Syst Rev. 2021;10(1):263.

5.	Munn Z., Peters M. D. J., Stern C., Tufanaru C., McArthur A., Aromataris E. Systematic review or scoping review? Guidance for authors when choosing between a systematic or scoping review approach. BMC Med Res Methodol. 2018;18(1):143.
